# Genetic Dissection of Root System Architectural Traits in Spring Barley

**DOI:** 10.3389/fpls.2019.00400

**Published:** 2019-04-02

**Authors:** Zhongtao Jia, Ying Liu, Benjamin D. Gruber, Kerstin Neumann, Benjamin Kilian, Andreas Graner, Nicolaus von Wirén

**Affiliations:** ^1^Molecular Plant Nutrition, Leibniz Institute of Plant Genetics and Crop Plant Research, Gatersleben, Germany; ^2^Genome Diversity, Leibniz Institute of Plant Genetics and Crop Plant Research, Gatersleben, Germany

**Keywords:** root trait, root architecture, quantitative trait locus, root observation box, rhizobox, GWAS

## Abstract

Breeding new crop cultivars with efficient root systems carries great potential to enhance resource use efficiency and plant adaptation to unstable climates. Here, we evaluated the natural variation of root system architectural traits in a diverse spring barley association panel and conducted genome-wide association mapping to identify genomic regions associated with root traits. For six studied traits, root system depth, root spreading angle, seminal root number, total seminal root length, and average seminal root length 1.9- to 4.2-fold variations were recorded. Using a mixed linear model, 55 QTLs were identified cumulatively explaining between 12.1% of the phenotypic variance for seminal root number to 48.1% of the variance for root system depth. Three major QTLs controlling root system depth, root spreading angle and total seminal root length were found on Chr 2H (56.52 cM), Chr 3H (67.92 cM), and Chr 2H (76.20 cM) and explained 12.4%, 18.4%, and 22.2% of the phenotypic variation, respectively. Meta-analysis and allele combination analysis indicated that root system depth and root spreading angle are valuable candidate traits for improving grain yield by pyramiding of favorable alleles.

## Introduction

Changing climate conditions and the need to reduce fertilizer input for the sake of environmental quality impose increasing constraints on agricultural plant production (Michael Beman et al., 2005; Liu X. et al., 2013). Besides further improvement of fertilization practices and plant management, exploitation of endogenous plant mechanisms and adaptation strategies gain importance in the development of resource-efficient crop cultivars. Therefore, current breeding approaches need to consider plant traits that allow cultivars to be developed able to overcome challenging growth conditions, such as transient periods of drought or suboptimal nutrient supplies. In this regard, root development plays a key role, as roots express a large range of highly variable physiological and morphological traits that favor water and nutrient uptake. More recently, root system architecture has received a particular increase in attention, as it determines the three-dimensional shape of the root system and thus the soil volume that can be explored for water and nutrients as well as yield formation (Giehl and von Wirén, 2014; Voss-Fels et al., 2018b).

It has proven highly promising to implement genetic information on root traits and root system architecture into breeding practice, whenever resource efficiency or stress tolerance needs to be enhanced. A considerable number of studies has shown that individual root anatomic or architectural traits correlate with enhanced nutrient uptake, water use efficiency or yield formation (Zhu et al., 2010; Postma and Lynch, 2011; Chen et al., 2014; Chimungu et al., 2014; Postma et al., 2014; Saengwilai et al., 2014; Mu et al., 2015; Liu et al., 2017; Robinson et al., 2018). At the genetic and molecular level, studies across several plant species have identified relations between early root traits and crop productivity or nutrient and water use efficiency (Tuberosa et al., 2002; Gamuyao et al., 2012; Uga et al., 2013; Hufnagel et al., 2014; Li et al., 2015). Such relations are confirmed by the co-occurrence of QTLs between seedling root traits and nutrient or water use efficiency or grain yield (Hamada et al., 2012; Christopher et al., 2013; Atkinson et al., 2015). In wheat, the drought-adapted genotype SeriM82 showed a more compact root system and allocated more roots to deeper soil layers, which was associated with a steeper seminal root angle and a larger number of seminal roots in seedlings (Manschadi et al., 2006, 2010). Scoring a diverse collection of elite durum wheat lines at different soil moisture levels for a genome-wide association study allowed 15 QTLs to be identified that overlap in traits of the seedling root system and in agronomic traits that may be relevant for overcoming drought (Canè et al., 2014). Assessing maize lines in hydroponic culture, Li et al. (2015) reported that 70% of nitrogen use efficiency (NUE)-related QTLs overlapped with those controlling seedling root traits, suggesting a large contribution of morphological root traits to NUE. An increase in grain yield was achieved by introgressing QTL clusters into advanced backcross-derived lines and testcrosses, providing direct evidence for the feasibility of improving grain yield by manipulating root systems (Li et al., 2015). In rice, Uga et al. (2011) discovered *Deep Rooting 1* (*DRO1*), a major locus controlling root growth angle, in a bi-parental population of two rice lines differing in their drought tolerance. Subsequent cloning and characterization of *DRO1* demonstrated its role in forming a steep root angle and consequently improving drought tolerance as well as grain yield when transferred into the genetic background of the susceptible parent (Uga et al., 2013). Furthermore, a direct genetic relation has been established between root length and P acquisition as well as yield performance in phosphorous-deficient soil using a certain allele of the *PHOSPHORUS STARVATION TOLERANCE 1* (*PSTOL1*) locus in rice or sorghum (Gamuyao et al., 2012; Hufnagel et al., 2014). Taken together, breeding new crop cultivars with superior root systems bears great potential to enhance resource use efficiency and plant adaptation to instable climates.

In the past, breeding efforts in crops have relied heavily on the monitoring and selection of above-ground traits that directly contribute to grain yield, whereas little attention has been paid to root architectural traits (Dunbabin et al., 2003; Den Herder et al., 2010). A major reason for the poor consideration of roots is the great difficulty in accessing root phenotypes when plants are grown in the field. Recently, roots of field-grown plants have been phenotyped by “shovelomics,” an approach in which root systems are dug out of the soil and scored visually for root traits (Trachsel et al., 2011). Although this approach works under crop production conditions and allows a reproducible scoring of genotypes for major root traits like root angle or crown and brace root number, it is restricted to plants with thicker roots. As an alternative, indoor culture systems allow a rapid, cost-effective and accurate quantitative evaluation of root systems from a large number of lines as required in genetic studies. Most indoor culture conditions use pots or boxes that restrict root phenotyping to early growth stages, raising the question whether root system architectural traits caught at early growth stages are predictive for root performance of mature plants grown in the field. To some extent, this assumption has been validated in wheat and maize, in which significant and positive associations were found between seminal root angle in the seedling stage and nodal root angle of adult field-grown plants (Ali et al., 2015; Maccaferri et al., 2016). Thus, these studies support the assumption that seminal root traits can be employed as promising proxies for the adult root system.

Barley is a major cereal crop providing essential raw material for malting and beer production, as animal feed and it also serves as an important staple crop in various countries. Like other cereals, barley plants have a typical fibrous root system consisting of seminal roots and post-embryonic nodal roots. The former originate directly from the embryo radicle, whereas the latter are formed at later developmental stages from lower tiller nodes (Wahbi and Gregory, 1995). Both root types develop lateral roots and root hairs, which are major components for nutrient and water absorption. Quantitative trait loci (QTL) mapping has provided valuable information on genomic regions controlling the genetic variation of root traits in barley (Chloupek et al., 2006; Naz et al., 2012, 2014; Arifuzzaman et al., 2014; Robinson et al., 2016, 2018), and most recently the flowering regulator *VRN1* has been implicated in regulating root system architecture (Voss-Fels et al., 2018a). Nonetheless, the genetic architecture controlling the barley root system and underlying molecular mechanisms have still remained unclear given the constraints of traditional QTL mapping, such as restricted allelic variation in examined gene pools or poor mapping resolution due to limited segregation and recombination (Zhu et al., 2008). Genome-wide association studies (GWAS), also known as linkage disequilibrium (LD) mapping, provide an alternative way to identify associations between quantitative values of phenotypic traits and molecular markers. In principle, GWAS takes advantage of the large number of historically and evolutionarily occurred recombination events and links these events with phenotype, allowing mapping at a more refined scale. In the present study, we employed a diverse panel of spring barley lines to estimate the natural variation of defined root traits for genome-wide association mapping to dissect the genetic basis for root system architecture. For this purpose, we grew plants in substrate-filled rhizoboxes that allow root spreading angle in parallel with other root traits to be recorded. This approach allowed identifying major QTLs for root system depth, root spreading angle and total seminal root length.

## Materials and Methods

### Plant Material and Genotyping

The spring barley collection used in present work consists of 221 accessions (148 cultivars, 55 landraces, and 18 breeding lines) selected from the Barley Core Collection (BCC) and the barley collection maintained of the Federal ex-situ genebank for Agricultural and Horticultural Crop Species maintained at the IPK Gatersleben, Germany. This panel has been described initially by Haseneyer et al. (2010) and used for genetic dissection of agronomic traits, salt tolerance, flowering time, tiller number and plant height (Haseneyer et al., 2010; Pasam et al., 2012; Long et al., 2013; Alqudah et al., 2014, 2016) and biomass accumulation in the two-rowed subset (Neumann et al., 2017). In detail, the association panel is composed of 125 two-rowed and 96 six-rowed accessions originating from 51 different countries and 4 geographical regions, Europe (*n* = 108), West Asia and North Africa (*n* = 45), East Asia (*n* = 40), and America (*n* = 28). Each accession has been propagated by single seed descent, amplified in the field and genotyped with a 9 K iSelect chip consisting of 7,864 SNPs as described by Comadran et al. (2012).

### Root System Phenotyping

**FIGURE 1:**
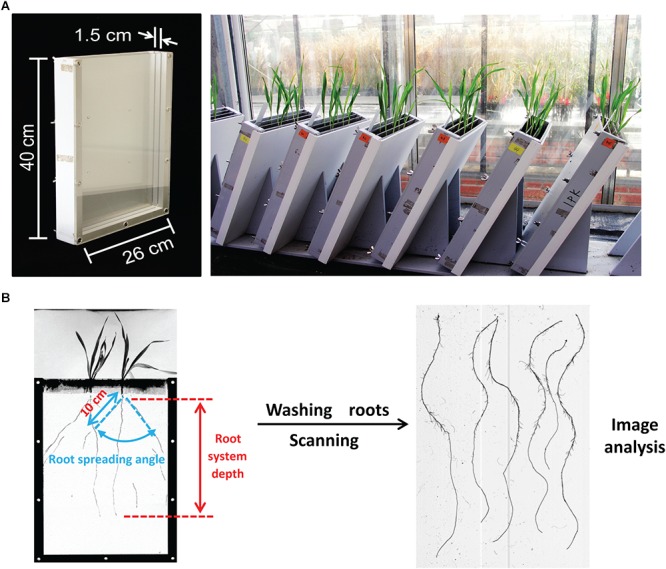
Root phenotyping and trait measurement. **(A)** Three-compartment root observation box and appearance of barley seedlings grown in the greenhouse for 12 days. **(B)** Schematic illustration of the procedure for root trait measurements.

Root phenotyping was conducted under well-controlled greenhouse conditions (long days, 16/8 h day/night and 20/16°C day/night). To increase the phenotyping capacity, we constructed rhizoboxes with three compartments, each (length ×°width ×°height = 26 ×°1.5 ×°40 cm) separated by transparent plexiglass plates and covered by the non-transparent plastic box to the outside (Figure 1A). Boxes were inclined by 60° to the horizontal plane with the plexiglass plate on the underside such that roots could grow along the surface. Plants were grown in a peat-based substrate (Klasmann Substrat 1) supplied with additional calcium and potassium (9 g/kg CaCO_3_, 5.04 g/kg CaO as well as 2 g/kg K_2_SO_4_). To ensure homogeneity, mineral salts were dissolved in distilled water before addition to the substrate. Each compartment was filled with 750 g substrate and then carefully supplemented with 220 ml distilled water to keep the same moisture. To avoid effects of germination on root growth, four seeds of a single genotype were sown directly in each compartment and thinned to 2 homogenous seedlings at 3 days after germination. Each genotype was sown in 3 different rhizoboxes, each carrying 2 plants per genotype, yielding 6 replicates per genotype. At 12 days after sowing, boxes were opened, and root traits were evaluated. In detail, the root system visible on the plexiglass surface was traced onto a transparent plastic sheet before each compartment of the box was opened. Then root system depth (RSD), i.e., the largest distance between the hypocotyl and the root tip, was measured with a scaled ruler. The root spreading angle (RSA) was defined as the angle between the two outmost seminal roots measured with a protractor at 10 cm below the hypocotyl (Figure 1B). The compartment of the box was then opened and the substrate was washed from the root system. The seminal root number (SRN) was counted manually, and roots were scanned (Epson Expression 10000XL at 300 dots per inch resolution while submerged in water) and quantified via image processing using the IAP software^1^ to determine TSRL, from which average seminal root length (ASRL) was calculated (TSRL divided by SRN). Shoots were dried at 65°C and weighed. Due to the large number of genotypes, the whole collection was phenotyped in 7 experimental batches, within each comprising 35–45 genotypes. The sixed-rowed spring cultivar Morex was repeated each time and served as an internal reference to account for batch effects.

### Population Structure and Linkage Disequilibrium (LD) Analysis

The population structure was estimated by principal component analysis using Eigenanalysis function implemented in Genstat v.16 according to Price et al. (2006). LD was analyzed in the whole germplasm collection by pair-wise comparisons among the SNP markers using TASSEL v.3.0 with default settings and presented with squared allele frequency correlations (*r*^2^) between the pairs of loci (Bradbury et al., 2007). The *r*^2^ values were plotted against the genetic distance between markers to estimate intra-chromosomal LD decay by fitting a smoothed LOESS curve in R (R Core Team, 2013). The critical *r*^2^ value was taken by the 95th percentile of the distribution of unlinked r^2^ referring to marker loci with map distance greater than 50 cM or located on independent linkage groups (Breseghello and Sorrells, 2006).

### Statistical Analysis and Association Mapping

Best linear unbiased estimates (BLUEs) of the root traits for each genotype were calculated based on the Restricted Maximum Likelihood (REML) method implemented in Genstat v.16 (VSN International). In the model, genotypes and replicates were fitted as fixed and random effects, respectively. Variance components were calculated based on REML where both genotype and replicate were treated as random effects. Broad-sense heritability (*h^2^*) was estimated as *h^2^* = σg^2^/ (σg^2^+ σe^2^/n), where σg^2^ is genotypic variance component, σe^2^ is residual variance component, n is replicates. The BLUE for each accession was used for computing pair-wise Spearman’s rank correlations among root traits and thousand kernel weight (TKW) collected from a field trial in 2012 using R (R Core Team, 2013). BLUEs were used for association mapping using the software TASSEL v.2.1 (Bradbury et al., 2007). Markers with minor allele frequency (MAF) of <5% and missing data >10% were filtered out, finally leaving 6336 informative markers. Initially, to select the most suitable statistical model, we made a model comparison by performing association analysis with two different statistical models, namely two mixed linear models (MLM) including kinship together with principle components (PCs) or kinship alone for correcting population structure. By evaluating the inflation of *p*-values through quantile-quantile (Q-Q) plot and GWAS results, we found that both mixed linear models yielded the same results, concluding that MLM including kinship is sufficient to control for population structure. To account for potential maternal effects of seed size on the association mapping of root traits, a third MLM was tested with kinship and TKW as a cofactor. However, the results from that model were almost identical to the MLM with kinship alone. Therefore, only associations resulting from the MLM with kinship are presented. The kinship model was performed based on the following equation: y = Xα+Kμ+e, where y is the phenotypic response vector, α is a vector of fixed effect for the marker to be estimated, K is the kinship matrix computed from TASSEL v.2.1, μ is the vector of random effect for co-ancestry, and e is the vector of residuals. A significant threshold of *P* < 0.1 correcting multiple tests with false discovery rate (FDR) (Benjamini and Hochberg, 1995) was applied to claim significant marker-trait associations. Considering the complexity of root traits, a second significance level –log_10_ (*P*-value) >3 was used in order to avoid ignoring minor effect loci and these QTLs were considered as suggestive QTLs. The allelic effect was computed as the difference between two alternative genotypes differentiated by the lead SNP with the minor allele serving as a base. The standard multiple regression approach described by Utz et al. (2000) was used to estimate the proportion of phenotypic variance explained by a single QTL (*R*^2^) and by all QTLs (adjusted *R*^2^).

### QTL Meta-Analysis and Candidate Gene Identification

To compare QTLs detected in the present study with those of previous work, QTLs underpinning root, shoot, phenology and agronomic traits in barley were collected from other studies (Pasam et al., 2012; Long et al., 2013; Alqudah et al., 2014, 2016, 2018; Wehner et al., 2015; Reinert et al., 2016; Robinson et al., 2016, 2018; Neumann et al., 2017). QTLs locating within a distance of 3.5 cM (according to the calculated average LD decay) were considered to be co-located. The QTLs detected in the present study and previously reported QTLs were projected into POPSEQ map and visualized with Mapchart v.2.3 (Voorrips, 2002; Mascher et al., 2013). In order to identify potential candidate genes underlying the QTLs, we systematically analyzed all genes located in the confidence interval and present in the barley genome database^2^. The most likely candidate genes were selected according to their function in root development as reported in Arabidopsis, maize, rice or *Brachypodium*.

## Results

### Population Structure and Linkage Disequilibrium Analysis

**FIGURE 2:**
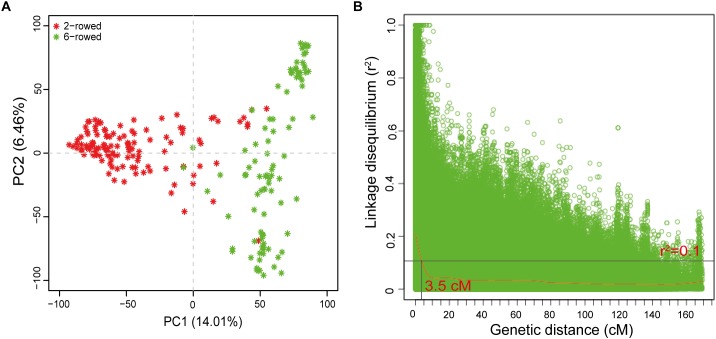
Population structure and LD decay of the spring barley collection. **(A)** PCA plot for population structure; **(B)** Intra-chromosomal LD (r^2^) decay of marker pairs over all chromosomes as a function of genetic distance (cM). The horizontal line indicates the 95th percentile distribution of unlinked *r*^2^. The loess fitting curve (red line) illustrates the LD decay.

Principle component analysis was used to estimate population stratification. The first two PCs successfully differentiated the population into two subgroups characterized by two- vs. six-rowed lines (Figure 2A). The intra-chromosomal LD decay was estimated to range from 1.7 cM for Chr 4H to 4.5 cM for Chr 3H (Supplementary Figure S1). The whole genome average LD decay was estimated to be 3.5 cM with a critical value of *r*^2^= 0.1 (Figure 2B). Therefore, further significant marker-trait associations within a distance of 3.5 cM were binned to a single QTL and presented with the most significant SNP.

### Phenotypic Analysis of Seminal Root Traits

Twelve days after sowing, a total number of 221 spring barley accessions grown in rhizoboxes were examined for seminal root traits (Supplementary Table S1). The broad-sense heritability values were moderate to high, ranging from 42.9% for ASRL to 84.9% for RSA, suggesting that the variation observed in root traits was under strong genetic control. Except for SRN, all root traits exhibited more than 2-fold differences (Table 1) with coefficients of variation (CV) from 18% to 26% for RSD and TSRL, respectively. In addition, all traits followed an approximately normal or normal distribution (Supplementary Figure S2), indicating a quantitative inheritance nature of root traits. Two subpopulations of the panel differed significantly in terms of population means for all traits (Supplementary Table S2). In general, 6-rowed barley showed smaller population means for RSD, TSRL, ASRL, SDW, and SRN, but exhibited a wider root angle than 2-rowed barley, indicating that 6-rowed barley has a relatively shorter and shallower root system than 2-rowed barley.

**Table 1:** Phenotypic variation and heritability for root and shoot traits of the spring barley collection.

Trait	Genotype no.	Min	Max	Mean ± SD	CV(%)	*h^2^*(%)
RSD	221	8.15	34.03	25.57 ± 4.5	17.60	84.7
RSA	221	46.67	131.67	70.5 ± 17.99	25.52	84.9
SRN	221	4.22	8.00	5.87 ± 0.9	15.33	72.7
TSRL	219	64.56	170.43	125.17 ± 32.44	25.92	54.4
ASRL	219	14.59	29.95	21.53 ± 5.45	25.56	42.9
SDW	221	18.05	70.13	45.9 ± 11.84	25.80	73.8


*Given are the total number of genotypes scored for each trait with its minimum and maximum values, the mean ± SD and the heritability (*h^2^*). RSD, root system depth; RSA, root spreading angle; SRN, seminal root number; TSRL, total seminal root length; ASRL, average seminal root length; SDW, shoot dry weight*.

Spearman’s rank correlations were calculated for root traits. As expected, positive and negative correlations were observed among root traits that are morphologically related (Supplementary Table S3). Strong to moderate positive correlations were found for TSRL and ASRL (*r* = 0.59), TSRL and SRN (*r* = 0.52), TSRL and RSD (*r* = 0.40), whereas a highly significant albeit slightly negative correlation was found between ASRL and SRN (*r* = -0.24). SDW was significantly and positively correlated with all root traits (*r* = 0.22–0.49), except for RSA that was negatively correlated (*r* = -0.13). Correlations were also calculated for thousand kernel weight (TKW) to determine whether kernel size had a major effect on scored traits. All of the root traits showed low to moderate correlations with TKW (*r* = -0.24–0.42, Supplementary Table S3).

### Association Mapping and Candidate Gene Identification Underlying QTLs

**FIGURE 3:**
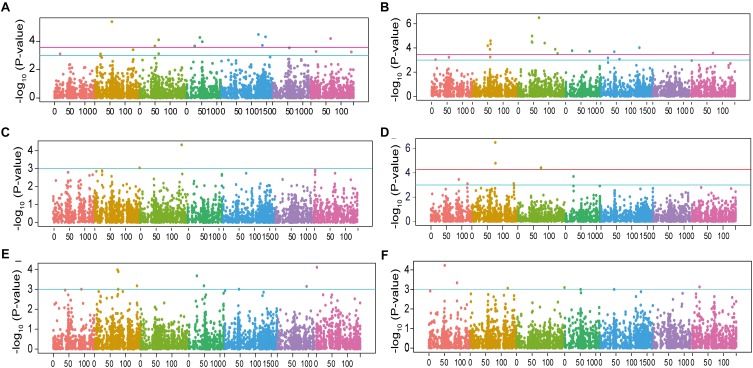
Manhattan plots for SNP association analysis of barley root traits. Negative Iog10-transformed *P*-values from genome-wide association scan were plotted against positions on each of the seven chromosomes of barley. Chromosomes are depicted in different colors (Chr 1H to Chr 7H, from left to right). The red and light-blue horizontal lines correspond to significance threshold FDR <0.1 and -Iog10 *P*-value >3, respectively. **(A)** Root system depth; **(B)** root spreading angle; **(C)** seminal root number; **(D)** total seminal root length; **(E)** average seminal root length, and **(F)** shoot dry weight.

**FIGURE 4:**
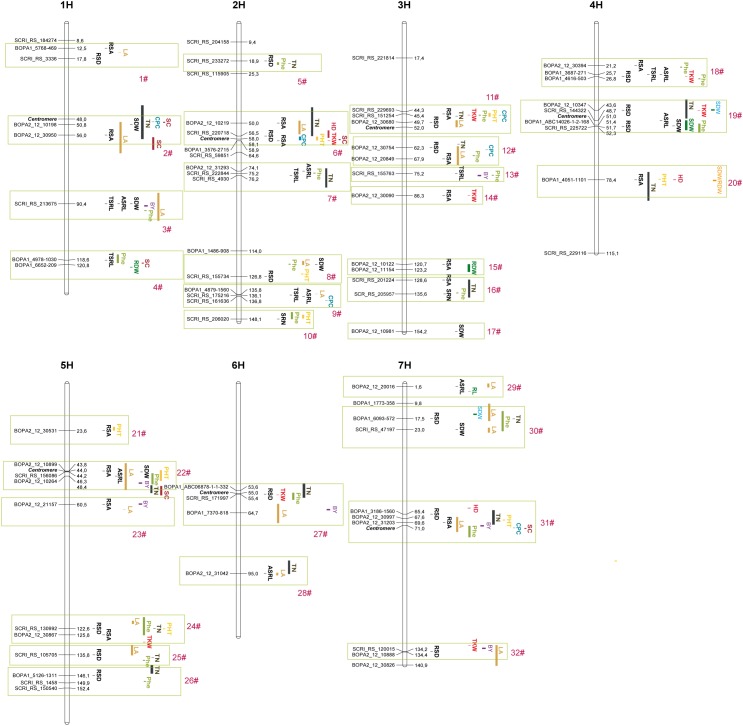
Location of quantitative trait loci (QTL, -log10*P*-value >3) projected on the barley POPSEQ map. Loci are presented with the most significantly associated markers. Single-component QTLs are reported as vertical bars corresponding to confidence interval or presented with the lead SNP reported in previous studies. HD, heading date; Phe, phenology; BY, biomass yield; TKW, thousand kernel weight; LA, leaf area; TN, tiller number; PHT, plant height, RDW, root dry weight; SC, starch content; CPC, crude protein content; RL, root length. Frames in light green highlight the QTL hotspots associated with root traits and agronomic traits.

In total, 65 marker-trait associations were found with a significant threshold of –log_10_ *P*-value >3 distributed over all 7 chromosomes with numbers varying from 2 on chromosome 6H to 11 on chromosome 2H (Figure 3, 4 and Supplementary Table S4). Among them, 30 marker-trait associations corresponded to a cut-off FDR <0.1 (Table 2). In a few cases, several close-by markers were binned into the same QTL based on the average LD decay of 3.5 cM, resulting in a total of 55 genomic regions including 25 QTLs (FDR <0.1) and 30 suggestive QTLs (–log_10_*P*-value >3 but FDR >0.1). These QTLs cumulatively explained between 12.1 and 48.1% of the phenotypic variance (Table 2). Of the six examined traits, RSD and RSA, showing the largest range of phenotypic variation and highest heritability values, were associated with the largest number of significant markers (Figure 3, 4, Table 1 and Supplementary Table S4). For RSD, a total of 10 significant and 6 suggestive QTLs were detected. The most significant association was found on Chr 2H (*qRSD3*, SCRI_RS_220718, 56.52 cM) with a –log_10_*P*-value = 5.39, contributing to 12.4% variation of RSD. The allele conferring longer RSD in this QTL increased RSD by 4.2 cm relative to the alternative allele at this peak. Amongst 16 loci detected for RSA, the most significant QTL mapped on Chr 3H (*qRSA6*, BOPA2_12_20849, 67.92 cM) explaining 18.4% of phenotypic variation. The major QTL accounting for 22.2% of phenotypic variation for TSRL was found on Chr 2H (*qTSRL3*, SCRI_RS_4930, 76.20 cM). In line with the positive correlation found between TSRL and ASRL, this QTL was also mapped for ASRL and explained 10.1% variation of this trait. For the traits SRN, ASRL, and SDW, there were only 2, 8, and 7 suggestive QTLs found, respectively. The most significant markers were located on Chr 3H (SCRI_RS_205957, 135.62 cM) for SRN, Chr 7H (BOPA2_12_20016, 1.63 cM) for ASRL and Chr 1H (BOPA2_12_10198, 50.85 cM) for SDW, respectively. Subsequent analysis of QTLs identified for each trait revealed QTL co-localizations, and 10 out of a total of 55 QTLs were found to be associated with more than one trait (Supplementary Table S5).

**Table 2:** Number of QTLs significantly associated with root and shoot traits and phenotypic variance explained (PVE).

Traits	QTL number	FDR <0.1	FDR 0.1>-log_10_*P*-value >3	PVE (*R*^2^_adjusted_%)
RSD	16	10	6	48.12
RSA	16	12	4	43.24
SRN	2	0	2	12.10
TSRL	6	3	3	33.85
ASRL	8	0	8	30.63
SDW	7	0	7	29.13


*FDR, false discovery rate; RSD, root system depth; RSA, root spreading angle; SRN, seminal root number; TSRL, total seminal root length; ASRL, average seminal root length; SDW, shoot dry weight*.

To explore the putative candidate genes underlying the identified QTLs, we systematically analyzed the annotated genes in close vicinity to significant SNP markers and identified a number of high priority candidate genes (Supplementary Table S6). For the most significant QTL detected for RSD (*q*RSD2, 2H, 56.52), we found several interesting candidate genes involved in flowering (*HvFT4*, *HvCEN*), sugar signaling (*HvSUSIBA2*) or phytohormone homeostasis (*HvGID2* and *HvARF6*). In addition, in this region we also found the known root development gene *HvSHR1*. In Arabidopsis and rice, SHR1 is crucial for maintenance of the root meristem (Helariutta et al., 2000; Cui et al., 2007). *HvARF15* appeared as a candidate gene for the most significant QTL associated with TSRL and ASRL (*q*TSRL3, 2H, 76.2, *q*ASRL2, 2H, 74.08). We further found that the QTL region 50.85–55.95 cM of 1H associated with SDW and RSA and contained the genes *HvAFB2*, *HvHXK1*, *HvGA2OX5*, *HvSUT2*, and *HvGID1-like*. One QTL (1H, 90.4) associated with SDW, TSRL, and ASRL mapped close to *HvHXK5*, *HvGA2OX4* and *HvARF4*. In rice, the locus *Deep Rooting 1* (*DRO1*) has been proven as a key determinant for root angle in the experimental population IR64 x Kinandang Patong (Uga et al., 2013). Its orthologous gene in barley was predicted to locate on Chr 5H (MLOC_3895.5, 48.38 cM) and mapped close to a QTL for RSA (*qRSA13*, Chr 5H, BOPA2_12_10899, 43.76 cM). In this region, we also found that the brassinosteroid biosynthesis gene *CPD* (MLOC_10658.1, 5H, 44.02 cM) associated with SDW (*q*SDW6, 5H, 44.17 cM) and ASRL (*q*ASRL6, 5H, 46.32 cM). In addition, an orthologous gene of *ZmRTH3* (MLOC_52864.1, Chr 4H, 52.33 cM) and *OsSCR1* (AK365059, Chr 4H, 51.41 cM) was found as promising candidate for *qRSD9* (SCRI_RS_225722, Chr 4H, 51.73 cM). One QTL for RSD (*q*RSD16, 7H, 134.20 cM) was mapped close to *CKX3* (7H, AK355215, 132.65 cM) and one QTL (4H, 21.2–26.7 cM) associated with RSA, RSD, TSRL and ASRL was mapped close to *HvINT-C* (MLOC_70116.1, 4H, 25.85 cM).

### Allele Combination Analysis for the Two Highly Heritable Traits RSA and RSD

**FIGURE 5:**
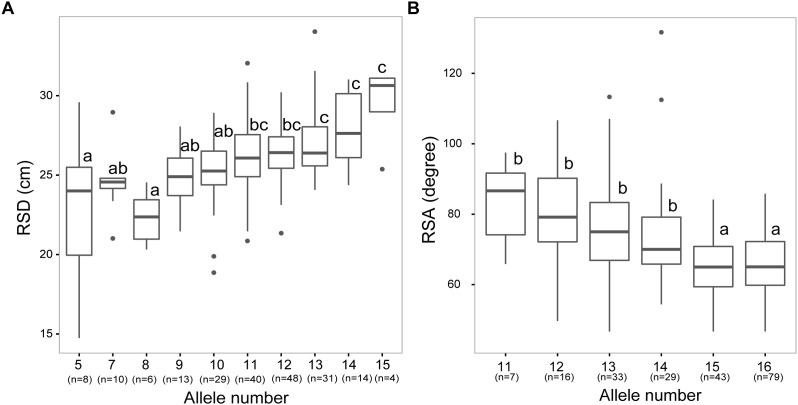
Combined allelic analysis reveals additive effects of root alleles. **(A,B)** Boxplot for trait values of genotypes with different number of alleles associated with longer root system depth (RSD; **A)** or narrower root spreading angle (RSA; **B)**. X-axis indicates the number of alleles carried by accessions, while numbers in brackets represent the number of genotypes in each group. Different letters indicate significant differences at *P* < 0.05 according to one-way ANOVA and post-hoc Tukey test.

In breeding approaches, high heritability of a root trait is one of the most important factors to successfully obtain an appreciable selection response for root system properties. Given the high heritability values of RSD and RSA (>80%) and their importance for adaptation to water or nitrogen deficit, both traits were selected in an attempt to assess whether they can be pyramided in breeding programs. We, therefore, grouped our germplasm collection according to allele combinations that were significantly associated with RSD and RSA (–log_10_*P*-value >3). Average RSD was significantly increasing with the number of alleles representing long RSD in the barley accessions. Most accessions possessed 10 to 13 alleles for long RSD, whereas only four accessions carried 15 alleles for long RSD (Figure 5A). Likewise, RSA for accessions with more than 15 alleles for narrow RSA was significantly narrower compared to that of sub-populations carrying a lower number of alleles for narrow RSA (Figure 5B).

## Discussion

Aiming at a deeper understanding of the genetic basis underlying the phenotypic variation in root system architecture, we conducted GWAS for defined root traits in a diverse population of spring barley genotypes. A total of 55 QTLs were detected that contribute to the genetic variation of the root architectural system in barley. Three major loci were detected on chromosome 2H and 3H, explaining a large part of the variation in root traits suitable for subsequent gene identification. Based on high heritability and cross comparisons with previously detected QTLs, allele combination analysis suggests that RSD and RSA are promising for marker-assisted selection (MAS) in breeding deep-rooting crop cultivars.

### Phenotypic Analysis of Root System Architectural Traits

The precise measurement of traits is a prerequisite for the success of genetic studies. Considering the technical difficulties encountered in the quantitative assessment of root traits from adult field-grown plants, quantifications of seminal root traits in plants grown in substrate-filled rhizotrons provide a reasonably fast and accurate alternative for phenotyping of hundreds of lines. Phenotypic evaluation of our spring barley collection in rhizoboxes revealed high heritabilities and a broad range of variation for individual root traits, in particular for RSD and RSA (Supplementary Figure S2 and Table 1). Unexpectedly, the variation for RSA determined in our panel was much smaller than that obtained from a much smaller population of elite barley lines (Robinson et al., 2016). This discrepancy may result in part from the fact that RSA was not measured in the same way. While Robinson et al. (2016) assessed the root angle between the first pair of seminal roots, we determined the angle between the two outmost seminal roots in soil-grown plants (Figure 1B), reflecting the maximum spreading angle achieved by seminal roots irrespective of their chronological appearance. In a previous study, root number of agar-cultivated wheat seedlings showed a strong correlation with grain yield of field-grown plants (Liu X.L. et al., 2013), supporting the idea that a larger number of seminal roots may provide superior early vigor that is particularly crucial for water uptake under drought-prone conditions (Richards, 2008; Reynolds and Tuberosa, 2008). In the present barley population, seminal root number exhibited a considerable variation, ranging between 4.2 and 8 (Supplementary Figure S2 and Table 1). This variation of almost factor 2 goes beyond the variation determined separately in wild barley, landraces or elite lines (Grando and Ceccarelli, 1995). Larger seminal root numbers in landraces and elite lines may be ascribed to domestication and breeding selection, which might have fixed the genetic variation of seminal root number at a higher level (Grando and Ceccarelli, 1995; de Dorlodot et al., 2007). For comparison, in the case of wheat, Canè et al. (2014) reported an even narrower variation between 4 and 6 seminal roots in a population of elite varieties.

Low to moderate correlations were found between root traits and TKW (*r* = -0.24–0.42, Supplementary Table S3). Indeed, similar correlations were also reported for other species such as maize (Pace et al., 2015) and wheat (Canè et al., 2014). Therefore, TKW was considered as a cofactor in the current QTL analysis. However, we obtained the same QTLs (data not shown), indicating a prevailingly independent genetic basis for the root traits and seed weight. Thus, such correlations may have very little impact on the identification of QTLs. A similar conclusion was reached by Canè et al. (2014). RSD and RSA, which are two opposing proxies for overall root depth, correlate positively and negatively with TKW, respectively (Supplementary Table S3). This type of correlation probably reflects a concerted morphological adaptation, in which deep rooting promotes water and nutrient uptake and consequently also grain filling.

### Association Mapping Identifies Three Major Loci Controlling Root Architectural Traits

Genetic studies on root volume and size have already been carried out with bi-parental populations (Chloupek et al., 2006; Naz et al., 2012, 2014; Robinson et al., 2016), and usually show less genetic variation and provide lower resolution for QTL detection (Zhu et al., 2008). Such poor resolution hampers the development of molecular markers for marker-assisted selection, particularly when close linkages exist with QTLs associated with undesired traits. GWAS offers a more refined mapping resolution that is largely determined by LD (Rafalski, 2010). In the present study, the average LD decay extended only to 3.5 cM, which is faster than that reported by Pasam et al. (2012), in which the LD decay was found to be 7 cM using the same barley panel but with a lower number of SNPs (Figure 2B). In this regard, the present study achieved higher resolution.

Employing a mixed linear model correcting for population structure using kinship, we were able to detect 55 QTLs distributed over all seven chromosomes (Figure 3, 4 and Supplementary Table S4). More than 80% of these QTLs, i.e., 46, explained less than 5% of the phenotypic variation (Supplementary Table S4), indicating that root traits are controlled by multiple loci with small effects. However, we detected three major QTLs, specifically *qRSD3* (Chr 2H, 56.52 cM), *qRSA6* (Chr 3H, 67.92 cM) and *qTSRL3* (Chr 2H, 76.2 cM) contributing between 12% and 22% to the variation of the respective root trait (Figure 3 and Supplementary Table S4) and highlighting the importance of these genomic regions in governing root growth. The number of detected QTLs was related to the phenotypic variability and heritability. RSA and RSD showed a large range of phenotypic variation at high heritability, and correspondingly, 16 QTLs were found for the respective traits. On the other hand, only two QTLs were found for SRN, a trait displaying less than two-fold variation (Supplementary Figure S2 and Tables 1, 2). In contrast to a study in a double haploid barley population, in which only two QTLs were mapped for root spreading angle (Robinson et al., 2016), our study detected 16 QTLs for RSA, cumulatively explaining more than 43% of the overall variation. This observation supports the advantage of GWAS over traditional linkage mapping. Comparing QTLs for different traits examined in the current work, a total of 10 genomic regions were found to associate with multiple traits (Supplementary Table S5). For instance, the genomic region on Chr 1H (90.4 cM) was associated with SDW, TSRL, and ASRL. All these traits are closely and significantly associated with each other (Supplementary Table S3). Other QTLs associated with multiple traits were located on Chr 2H, 4H, 5H, and 7H (Supplementary Table S5). The co-localization of these QTLs supports the correlation identified among traits (Supplementary Table S3), suggesting potential pleiotropic effects or linkage of the genetic constituents of these traits.

### Genetic Association of Seedling Root Traits With Agronomic Traits

Although the advantage of a superior root system has long been recognized, the genetic association between the barley root system and agronomic traits remains to be established. Recently, Robinson et al. (2016) evaluated the genetic relationship between seminal root traits and agronomic traits and found that plant height was correlated neither with root angle nor with root number, while the genetic association between root traits and grain yield depended largely on the growth context (Robinson et al., 2018). We found that 15 QTLs for plant height, 7 for crude protein content, 7 for starch content, 32 for flowering time and 8 for TKW co-localized with QTLs for those root traits that we determined. In addition, we found 9 QTLs for shoot mass and 2 for root mass at the vegetative phase that co-located with root QTLs detected in the present study. Additionally, 29 and 27 QTLs for tiller number and leaf area, respectively, mapped together with root QTLs (Figure 4 and Supplementary Table S4). These findings provide strong evidence for the genetic association of early root traits and agronomic traits. A comparative analysis of QTLs associated with agronomic traits and root traits revealed 32 QTL hotspots distributed across 7 chromosomes (Figure 4). Eight QTL clusters (6#, 11#, 14#, 18#, 19#, 24#, 27#, and 32#) appeared of particular interest based on their co-location and the concomitant allelic effect on TKW. Each of these 8 clusters harbors at least one QTL associated with RSD or RSA (Figure 4 and Supplementary Table S4). The allele conferring longer RSD and the allele conferring narrower RSA may concomitantly increase TKW, delay flowering time and increase starch content while decreasing crude protein content (Figure 4 and Supplementary Table S4). Such positive associations suggest that a deep rooting phenotype may boost vegetative plant growth and finally contribute to grain filling.

### Putative Candidate Genes for Root-Associated QTLs

Our analysis indicated that several of the identified QTLs for root traits associated with genes involved in the synthesis, transport and signaling of sugars or phytohormones (Supplementary Table S6). Gibberellins and auxin have been shown to be essential for proper root development. For the major locus associated with RSD (*q*RSD3), we found the gibberellin receptor *HvGID2* (MLOC_61457.1) underlying this locus. A previous study in Arabidopsis has shown that mutants lacking GID-type GA receptors develop significantly shorter roots (Griffiths et al., 2006). Another two candidates likely underlying this locus are *HvARF6* (MLOC_64596.1) and *HvAFB2* (MLOC_56088.1) that are both involved in auxin signaling. *SUSIBA2* is a transcriptional activator in sugar signaling, and transgenic rice expressing barley *SUSIBA2* altered sugar allocation in a way that root growth was significantly reduced (Su et al., 2015). Thus *SUSIBA2* (AK369730) appears also as a candidate gene for *q*RSD3. An association between root traits and sugar-related processes is also supported by the co-localization of QTLs for starch content and root growth (Figure 4 and Supplementary Table S4). Interestingly, our GWAS results further suggest a conserved role of the *SHR*-*SCR* regulatory module in the root development of barley. Two QTLs for RSD (*qRSD3*, Chr 2H, 56.52 cM and *qRSD9*, Chr 4H, 51.73 cM) were co-localized with barley genes corresponding to *OsSHR1* (MLOC_62665.1, Chr 2H, 58.06 cM) and *OsSCR1* (AK365059, Chr 4H, 51.41). In Arabidopsis, *SCARECROW* (*SCR*) is specifically expressed in the endodermal cell layer of roots and activated by the transcription factor *SHORTROOT* (*SHR*), which is expressed in the stele but moves out to regulate endodermal differentiation (Di Laurenzio et al., 1996; Cui et al., 2007). Mutations in either gene causes short roots. The same functional role as in Arabidopsis was also found for *OsSCR1* and *OsSHR1* in rice (Kamiya et al., 2003; Cui et al., 2007; Mai et al., 2014). The auxin response factor *ARF15* (AK364144) appeared as candidate underlying the most significant locus associated with TSRL and ASRL (*q*TSRL3, 2H, 76.2 cM; *q*ASRL2, 2H, 74.08 cM). The major QTL associated with RSA (*q*RSA6, 3H, 67.92 cM) mapped to close *HvCCD8* (MLOC_66551.1) and *HvGA2O × 1* (AK364775), which are involved in strigolactone and gibberellin metabolism, respectively. A significant QTL (*q*RSA13) was mapped close to the gene MLOC_3895.5 (Chr 5H, 48.38 cM) orthologous to *DRO1* that largely determines root angle in rice (Uga et al., 2011, 2013). A near-isogenic line carrying the corresponding allele in rice showed elevated drought tolerance and higher yield under water deficit. However, in our study this QTL was not the most significant one for RSA, suggesting that *DRO1* might not be a major determinant of RSA variation in the present association panel. In fact, resequencing of *DRO1* in a natural population of rice did not identify the 1 bp-deletion that causes shallow rooting of cultivar IR64, suggesting that this mutation might be a rare allele (Lou et al., 2015). In addition to association with RSA, this region was also detected for SDW (*q*SDW6, 5H, 44.17 cM) and ASRL (*q*ASRL6, 5H, 46.32 cM). It is unlikely that *DRO1* plays a pleiotropic role in controlling SDW and RSD, as it was reported that *DRO1* only impacts root angle but has no pronounced effect on root length as well as shoot growth (Uga et al., 2013). We found that *HvCPD* (MLOC_10658.1, 4H, 44.02 cM) is a likely candidate underlying these two QTLs. *CPD* encodes a key enzyme in brassinosteriod biosynthesis, and plants deficient of brassinosteriods exhibit significantly lower biomass and shorter roots (Szekeres et al., 1996). In maize, *ZmRTH3* belongs to a monocot-specific clade of the *COBRA* gene family, and the corresponding mutant *rth3* properly initiates root hairs but fails to elongate these (Hochholdinger et al., 2008). We mapped a QTL (*qRSD*9, Chr 4H, 51.73 cM) in proximity to its orthologous gene (MLOC_52864.1, Chr 4H, 52.34 cM). Recently, a candidate gene-based association approach found that natural allelic variations in *ZmRTH3* were directly associated with seedling root growth under different N regimes and even with grain yield (Kumar et al., 2014; Abdel-Ghani et al., 2015), reinforcing a crucial role of *ZmRTH3* in root development and yield formation. *TB1* was shown to be essential for lowering tiller number during maize domestication, and its expression level positively correlates with root growth (Gaudin et al., 2014). One QTL region associated with RSD, ASRL, RSA, and TSRL (4H, 21.2–26.77) mapped close to the orthologous barley gene *HvINT-C* (MLOC_70116.1, 4H, 25.85 cM). More recently, enhanced cytokinin degradation as achieved by root-specific expression of a cytokinin oxidase (*CKX*) gene in barley significantly improved root growth and tolerance to drought stress (Ramireddy et al., 2018). We found that *CKX3* (AK355215, 7H, 132.65) is likely underlying one QTL associated with RSD (*q*RSD16, 7H, 134.20 cM). Taken together, our GWAS identified a number of high priority candidate genes that could be useful for future sequence mining and marker development for marker-assisted selection.

### Co-localization of Root-Associated QTLs With Genes and QTLs Involved in Flowering

Flowering time is an important adaptive trait and is considered as one important goal when breeding for high grain yield. Recently, it has been reported that during maize domestication selection on flowering time had a considerable impact on nodal root formation, as approx. 50% of the detected loci for nodal root number overlapped with QTLs for flowering time (Zhang et al., 2018). In the present GWAS, we also found that approx. 50% of root growth QTLs co-localized with known flowering loci (Figure 4 and Supplementary Table S4; Pasam et al., 2012; Alqudah et al., 2014). *q*RSA15 (Chr 5H, 125.7 cM) co-located with the flowering gene *VRN-H1*. Very recently, Voss-Fels et al. (2018a) reported that polymorphisms in *VRN-H1* modulate root growth angle and root length at both early and mature stages in wheat and barley, raising the possibility that *VRN-H1* might be the causal gene for this region. Moreover, QTLs associated with RSD and RSA (*qRSD2*, Chr 2H, 18.91 cM; *qRSD3*, Chr2H, 56.52 cM; *qRSA4*, Chr 2H, 58.92 cM) were located in close proximity to the genes *Ppd-H1* (Chr 2H,19.9 cM) and *HvCEN* (Chr 2H, 58.0 cM) that play a central role in regulating flowering time in barley (Turner et al., 2005; Comadran et al., 2012). In accordance, Arifuzzaman et al. (2016) also reported that the heading date gene *Vrn-H3* significantly associated with shoot and root biomass, although in our panel no QTL was detected in its vicinity. We assume that selection and breeding may have indirectly selected for root traits when selecting for favorable flowering time and high grain yield. Whether the close genetic association between flowering time and root traits indicates a causal relationship remains open, because individual genes determining either trait could just be in close genetic linkage. Recently, Voss-Fels et al. (2018b) proposed a context-specific interaction between flowering time point and root development, in which early flowering lines with deeper root systems gain yield rewards especially under terminal drought. Assessing root system architecture in existing near-isogenic and mutant lines of flowering time genes will contribute to a better understanding of the interaction and trade-off for biomass partitioning between roots and shoots and thereby help to design root systems in modern crops that are better adapted to fluctuating environmental conditions.

### Potential for Pyramiding RSD and RSA Alleles in Future Breeding Approaches

The depth and angle of a root system are the major determinants for the soil volume that can be explored by the root system. In the present study, 4 RSA and 6 RSD loci co-located with QTLs for TKW as already detected by Pasam et al. (2012), suggesting an important contribution of deep root growth to grain filling. In particular, we found that in 11 RSD and RSA QTLs (*q*RSD3, *q*RSD5, *q*RSD7, *q*RSD8, *q*RSD9, *q*RSA4, *q*RSA6, *q*RSA7, *q*RSA11, *q*RSA12, and *q*RSA15) alleles conferring longer RSD and narrower RSA increased TKW by more than 5 g (Supplementary Table S4). Consistent with our work, a previous study in durum wheat also revealed seedling root QTLs affecting agronomic performance and grain yield (Canè et al., 2014; Maccaferri et al., 2016). In an attempt to assess the potential of marker-assisted selection of root traits, especially RSD and RSA in future breeding, we grouped our genotypes according to their allelic state at each of the 16 RSD and 16 RSA loci. Genotypes with 15 beneficial alleles of RSD loci showed a deeper rooting phenotype than other subsets (Figure 5A). The majority of lines in the examined collection bear 10 to 13 favorable alleles, whereas only 4 lines carry more than 15 alleles, revealing that there is still potential for pyramiding these alleles in breeding practice. A similar additive effect was also observed for RSA. However, unlike RSD, the majority of lines (58% of the whole panel) carry more than 15 favorable alleles for the 16 RSA QTLs, resulting in even steeper roots (Figure 5B). Interestingly, we observed a further increase up to 80% of the 122 improved barley cultivars that possess more than 15 favorable alleles for RSA. This enrichment of beneficial alleles in the modern lines is most likely a result of breeding selection. In the past decades, yield increase through breeding has been associated with improvement of traits like earlier flowering or reduced plant height, which in turn could have inadvertently resulted in the selection of more efficient root systems (Paez-Garcia et al., 2015). The present study suggests that in particular RSA made a substantial contribution to this development, because a much larger number of lines carried multiple favorable alleles for RSA than for RSD (Figure 4). Consistent with this, de Dorlodot et al. (2007) reported that modern barley cultivars had been selected for a better spatial arrangement of roots compared with their wild progenitors. The results shown here also point to the option that future breeding programs take wild barley accessions into consideration for better exploiting exotic alleles to optimize root spreading angle. In support of this conclusion, Sayed et al. (2017) recently reported that wild barley ISR42-8 showed a steeper root system than the elite cultivar Scarlett with more roots allocated to deeper soil layers.

## Conclusion and Perspectives

This study evaluated root traits in 221 barley lines and in combination with GWAS three major loci were detected and several high priority candidate genes were identified. In follow-up studies, candidate gene-based resequencing and functional characterization will be necessary to elucidate the role of these genes in root development. Furthermore, root system depth and root angle showed highest heritability values and overlapped with QTLs for thousand kernel weight, thus appearing as the most promising root traits for marker-assisted selection and breeding deep-rooting crop varieties that are better adapted to water- and nitrogen-limited environments. Apart from studying seminal roots, nodal roots also play an important role in plant productivity, as indicated by the genetic association between nodal root angle and leaf longevity as well as grain yield in sorghum (Mace et al., 2012). This points to the necessity of phenotyping separately seminal and nodal root traits in dependence of plant development. Future approaches directly assessing the variation of seminal and nodal root traits in relation to yield components under varying field conditions, i.e., with respect to rainfall or nutrient supply, would help gaining a comprehensive view on the “QTLome” for root systems and their relation to yield formation in barley.

## Author Contributions

BG, AG, and NvW conceptualized the research. BG and YL conducted the phenotyping. ZJ, KN, and NvW analyzed the data. ZJ, KN, BK, AG, and NvW wrote the manuscript.

## Conflict of Interest Statement

The authors declare that the research was conducted in the absence of any commercial or financial relationships that could be construed as a potential conflict of interest.

## Footnotes

1 https://openimageanalysisgroup.github.io/IAP/

2 http://webblast.ipk-gatersleben.de/barley/
