# Supplementary material for: Genetic Dissection of Root System Architectural Traits in Spring Barley
Source: Front Plant Sci. 2019 Apr 2;10:400. doi: 10.3389/fpls.2019.00400 (PMC6454135; doi:10.3389/fpls.2019.00400)
Supplement: Supplementary file 1 [file Data_Sheet_1.pdf]

Supplementary Material

Genetic dissection of root system architectural traits in spring barley

Zhongtao Jia[1], Ying Liu[1], Benjamin D Gruber[1#], Kerstin Neumann[2], Benjamin Kilian[2##], Andreas Graner[2], Nicolaus von Wirén[1*]

* Correspondence: Nicolaus von Wirén,

E-mail: vonwiren@ipk-gatersleben.de

Table S1. List of spring barley genotypes used in this study with phenotypic values. RSD, Root system depth; RSA, Root spreading angle; SRN, Seminal root number; TSRL, Total seminal root length; ASRL, Average seminal root length; SDW, Shoot dry weight. NA, not available.

Genotype	Row number	RSD	RSA	SRN	SDW	TSRL	ASRL
BCC003	2	29.57	68.66	6.58	46.52	167.8	25.23
BCC093	2	30.73	68.66	6.095	52.92	165	26.79
BCC126	6	27.92	74.32	5.419	35.78	117.1	23.24
BCC129	6	28.39	91.28	6.58	49.21	148.7	21.24
BCC131	6	24.68	80.68	6.095	47.4	116.5	19.37
BCC1367	2	25.63	83.51	5.732	43.23	NA	NA
BCC1368	2	26.08	79.26	5.489	44.26	115.3	21.36
BCC1370	2	24.51	70.05	6.39	49.6	138.2	20.63
BCC1371	2	26.89	65.84	5.97	43.74	124.7	21.93
BCC1372	2	23.33	73.1	6.406	40.86	138.6	20.77
BCC1373	2	24.74	62.3	5.853	41.35	126.4	21.97
BCC1374	2	25.75	67.15	5.695	50.72	126.2	22.94
BCC1376	2	26.38	63.72	5.853	48.57	135.9	23.3
BCC1377	2	24.89	72.9	5.368	49.27	127.1	24.29
BCC1378	2	23.81	57.35	5.732	42.83	120.9	21.29
BCC1379	2	25.26	73.61	5.853	49.53	121.4	20.77
BCC1380	2	27.65	73.61	5.974	60.53	148.2	24.72
BCC1381	2	25.65	65.1	5.714	40.82	112	19.63
BCC1382	2	24.3	62.77	6.216	43.71	128.2	20.2
BCC1383	2	24.98	67.25	5.368	52.34	112.5	22.1
BCC1384	2	22.2	75.02	5.732	51.84	116.2	20.73
BCC1385	2	25.2	76.82	6.227	41.46	123.5	19.64
BCC1386	2	24.85	62.37	6.146	47.37	122.3	19.75
BCC1387	2	26.21	72.53	5.901	49.24	132.5	22.5
BCC1389	2	25.14	54.53	5.97	45.89	133	22.19
BCC1390	2	26.87	80.95	5.974	44.86	128.4	21.85
BCC1391	2	24.29	81.79	5.984	45.05	125	20.81
BCC1392	2	25.54	67.25	5.853	42.76	122.5	21.02
BCC1394	2	26.01	55.94	6.246	49.85	139.5	21.59
BCC1395	2	24.64	59.47	5.732	44.66	115.1	20.26
BCC1396	2	25.18	64.42	6.095	47.8	140.3	22.86
BCC1397	2	24.22	58.06	6.337	51	131	19.95
BCC1398	2	26.11	70.78	5.974	53.12	121.3	20.33
BCC1399	2	25.84	82.09	5.853	51.64	131.8	23.29
BCC1400	2	26.98	61.6	6.337	48.14	129.9	19.87
BCC1401	2	25.44	72.9	5.61	48.39	123.5	22.66
BCC1402	2	25.63	66.54	6.216	46.15	131.6	20.7
BCC1403	2	27.8	79.97	5.732	52.67	125.3	22.11
BCC1404	2	25.53	70.08	5.974	51.98	133.5	22.41
BCC1405	2	26.48	70.78	5.974	46.97	136.5	22.75
BCC1407	2	22.54	67.25	5.974	46.64	132.2	22.02
BCC1408	2	25.55	61.86	5.974	37.85	121.1	20.17
BCC1409	2	27.4	70.78	6.216	54.17	137.6	21.7
BCC1410	2	26.32	61.03	5.853	49.41	131.8	22.37
BCC1411	2	24.89	66.8	6.458	51.01	139.9	21.12
BCC1412	2	25.35	70.78	6.337	51.24	147.7	22.76
BCC1413	2	30.12	77.14	5.853	50.31	143.5	24.85
BCC1414	2	25.83	82.09	5.61	39.27	113.4	20.69
BCC1415	2	25.28	68.66	5.832	44.96	113.1	21
BCC1416	2	26.92	72.9	5.97	44.95	140.3	22.28
BCC1417	2	24.53	77.85	5.368	48.49	118.4	22.96
BCC1418	2	29.58	65.84	5.732	50.74	136.6	24.14

BCC1419	2	25.81	50.29	6.216	48.46	130.4	21.81
BCC1420	2	24.79	65.84	5.853	46.05	127.9	21.9
BCC1421	2	27.55	61.6	5.368	50.48	133	25.67
BCC1422	2	24.8	63.01	6.337	47.41	126.2	19.38
BCC1423	2	26.31	68.66	5.732	43.89	129.8	22.78
BCC1424	2	24.93	69.6	5.982	38.83	115.9	19.27
BCC1425	2	22.01	73.61	6.246	46.13	125	19.68
BCC1428	2	24.99	63.6	6.436	44.03	126.1	19.38
BCC1430	2	26.77	62.78	6.476	46.22	132.8	20.27
BCC1431	2	26.83	60.18	5.974	55.61	144.6	24.22
BCC1432	2	25.61	65.13	5.368	48.5	125.4	23.22
BCC1433	2	24.5	64.3	6.055	51.26	126.8	20.24
BCC1439	2	26.41	73.61	6.822	53.64	162.5	22.65
BCC1440	2	25.67	66.54	7.245	51.2	152.4	20.43
BCC1441	2	27.57	59.47	6.216	51.6	137.8	21.69
BCC1442	2	25.38	57.41	6.227	65.55	133.9	21.41
BCC1443	2	24.8	68.66	6.458	56.06	132.6	20.02
BCC1444	2	26.17	70.08	5.966	53.07	126.7	22.66
BCC1445	2	27.03	65.13	5.974	54.52	135.7	22.61
BCC1447	2	27.86	55.23	6.216	51.92	147.8	23.2
BCC1448	6	21.38	68.8	5.845	41.03	93.8	16.08
BCC1450	6	23.05	64.25	6.21	42.7	115.6	18.39
BCC1452	6	24.87	76.44	6.095	46.81	123.5	20.02
BCC1453	6	25.91	66.54	6.384	49.48	132.3	20.73
BCC1455	6	24.03	63.01	5.489	53.6	117.2	21.98
BCC1456	2	24.24	60.89	5.974	46.66	116.8	19.48
BCC1457	2	25.08	77.14	6.337	56.31	150.9	23.24
BCC1458	2	24.33	83.51	6.216	48.98	114.4	18.51
BCC1459	2	27.26	67.96	5.974	58.3	147.8	24.64
BCC1461	2	27.96	52.41	6.58	57	152.3	21.2
BCC1463	2	26.79	54.34	5.489	47.68	120.2	23.16
BCC1465	2	22.11	70.78	6.58	44.72	134.1	19.57
BCC1466	2	24.86	68.66	5.61	50.28	116.3	21.24
BCC1467	2	26.42	78.56	5.974	54.9	150.6	25.09
BCC1468	2	24.76	58.77	6.337	50.07	121.8	18.93
BCC1469	2	25.58	60.18	6.337	49.66	115.4	17.8
BCC1470	2	29.4	60.89	5.489	64.03	141.9	26.69
BCC1471	2	27.18	53.82	6.216	54.01	154.2	24.25
BCC1472	2	24.34	59.9	5.974	42.03	106.2	17.94
BCC1474	6	30.09	59.47	5.646	54.85	142.2	26.14
BCC1476	6	25.48	70.08	5.974	49.85	127.8	21.3
BCC1479	6	24.86	59.47	5.732	43.15	111.8	19.85
BCC1480	2	25.28	63.01	6.797	51.42	141	18.55
BCC1481	2	25.94	73.33	6.337	51.9	131.5	20.38
BCC1482	2	26.38	53.11	6.822	44.31	139.8	19.52
BCC1483	2	25.31	65.13	6.605	49.81	123.2	18.68
BCC1484	6	24.67	79.97	5.974	47.67	128.3	21.42
BCC1485	6	26.88	70.08	5.966	45.85	130.5	21.67
BCC1487	2	25.56	67.96	6.095	47.95	139.5	22.59
BCC1488	6	22.38	70.09	5.61	40.87	93.2	17.01
BCC149	6	27.94	91.28	5.974	43.36	121.6	20.53
BCC1490	6	23.56	81.39	6.58	47.11	141.6	20.85
BCC1491	6	23.75	65.55	6.095	38.79	105.7	17.31
BCC1493	6	26.2	52.24	5.073	43.29	99.9	20.51
BCC1494	6	25.84	70.08	6.095	43.66	132.8	21.67
BCC1497	2	26.25	53.82	5.247	52.8	112.7	22.55
BCC1498	6	24.33	53.82	5.732	45.86	115.67	19.78
BCC1500	6	23.04	70.09	5.97	49.55	115.7	19.43
BCC1503	6	26	74.32	5.489	53.38	118.1	22.09

BCC1504	6	25	71.91	5.853	45.01	115.8	19.89
BCC1505	6	27.28	53.11	5.646	47.33	118.6	20.05
BCC1506	2	28.81	55.94	6.095	46.15	139.2	22.7
BCC1524	2	25.74	67.25	6.822	46.99	142.3	20
BCC1529	6	25.38	70.78	5.732	44.62	120.2	21.63
BCC1541	2	27.59	58.77	5.853	50.45	120.3	20.68
BCC1548	6	24.98	60.18	6.216	55.2	134.6	21.13
BCC1561	6	26.09	67.62	5.61	47.02	126.5	22.72
BCC1565	6	26.85	67.96	5.853	48.39	120.9	20.83
BCC1566	2	25.33	63.72	6.458	51.23	135.1	20.09
BCC1589	2	25.45	61.6	5.974	47.51	123.7	20.61
BCC161	6	26.59	82.8	5.61	41.95	122	24.41
BCC167	6	30.81	101.18	5.966	46.36	114.2	18.04
BCC173	6	24.37	93.4	5.974	44.28	123.3	21.95
BCC182	6	24.33	65.13	6.095	34.43	110.6	18.43
BCC190	2	29.27	68.66	5.126	45.03	142.4	29.94
BCC192	2	25.9	67.15	5.656	39.62	105.2	18.78
BCC195	2	29.06	68.8	6.227	45	148.7	23.62
BCC197	2	28.56	53.11	5.005	46.11	120.9	26.15
BCC218	6	21.04	57.35	4.884	34.38	72.4	14.59
BCC219	6	19.89	71.74	5.247	37.65	127.3	28.61
BCC421	6	26.32	50.29	6.108	38.5	111.5	17.04
BCC423	6	21.25	61.62	6.361	48.68	110.8	16.39
BCC427	2	24.76	67.25	6.58	50.65	NA	NA
BCC432	2	26.45	69.37	5.695	40.94	118.2	22.83
BCC434	2	21.61	81.61	6.216	45.32	144.2	26.25
BCC436	6	21.14	86.33	6.149	42.93	127.7	20.43
BCC438	6	26.76	77.14	6.216	37.6	124.2	19.44
BCC439	6	24.12	68.69	6.146	43.92	106.5	17.32
BCC445	6	24.67	75.02	5.368	37.29	123.1	25.5
BCC446	6	22.57	82.8	5.557	39.08	127.2	23.43
BCC447	6	19.96	88.56	6.369	44.96	114.6	18.43
BCC502	6	29.06	74.32	5.974	41.29	133.5	23.28
BCC524	6	27.05	58.77	5.974	36.08	133.8	21.76
BCC526	6	33.32	83.3	5.33	43.75	139.1	27.08
BCC527	6	29.45	89.87	5.853	54.85	129.4	22.81
BCC532	6	26.28	79.26	5.61	51.83	112.6	20.39
BCC533	6	25.7	67.25	5.695	35.7	86.5	14.84
BCC535	6	15.77	87.02	5.494	42.6	116.6	21.76
BCC538	6	27.57	64.42	5.368	39.13	116.6	22.74
BCC551	6	29.28	50.29	5.005	44.4	109.7	23.93
BCC577	6	25.38	85.63	6.095	46.77	118	19.66
BCC579	6	27.6	79.26	6.458	41.82	117.3	17.69
BCC581	6	26.28	67.25	5.853	37.78	114.7	19.56
BCC625	6	25.16	78.56	5.247	42.12	95.9	19.18
BCC642	6	28.51	122.38	5.732	49.71	120.3	21.36
BCC666	6	26.79	106.12	5.853	44.39	117.4	20.82
BCC667	6	22.11	106.83	5.126	44.55	92.2	18.99
BCC675	2	27.25	71.49	5.732	48.12	122.6	21.71
BCC695	6	26.07	83.51	5.126	47.23	106.9	22.06
BCC718	6	12.53	83.59	5.984	42.56	118.2	20.46
BCC719	6	12.87	94.11	5.61	47.74	93.6	16.94
BCC729	6	9.65	125.15	4.922	23.03	64.6	15.52
BCC732	6	27.19	79.14	5.853	39.59	122.4	20.9
BCC745	6	27.29	89.87	5.732	43.69	123.6	21.99
BCC759	6	24.59	73.66	5.819	45.05	112.2	19.58
BCC761	6	27.12	66.54	5.732	42.68	122.6	21.39
BCC766	6	26.04	74.2	5.489	45.79	123.9	23.18
BCC768	6	26.28	94.78	5.97	41.36	117.2	20.56

BCC776	6	27.66	101.88	5.732	42.07	116.7	20.86
BCC801	2	26.89	66.54	4.884	39.53	103.6	22.72
BCC806	6	26.45	94.11	5.368	42.25	117.1	22.63
BCC807	6	27.53	70.08	6.095	45.39	133.5	21.72
BCC812	2	29.6	63.72	5.732	47.93	137.1	24.16
BCC814	6	26.74	79.26	4.762	42.73	90.2	20.58
BCC817	6	29.78	83.26	5.966	46.67	132.8	19.63
BCC818	6	26.81	79.26	5.368	41.77	123.5	23.42
BCC844	6	20.77	79.14	5.419	46.51	100.4	19.35
BCC846	6	30.34	70.08	5.247	54.93	130.8	26.27
BCC847	2	30.28	55.94	6.095	43.78	147.5	23.68
BCC852	6	29.18	75.02	6.216	50.35	135.5	21.38
BCC857	6	25.9	82.09	5.61	44.53	113.4	20.5
BCC860	2	29.74	66.8	6.108	47.2	131.7	21.21
BCC861	2	28.63	77.85	6.797	42.9	156	23.6
BCC868	6	26.76	79.97	5.61	42.56	116.8	21.49
BCC875	6	25.55	71.74	5.695	44.18	128	22.93
BCC881	6	25	70.78	5.489	39.57	94.6	17.77
BCC888	6	23.76	62.01	5.774	40.59	104.6	18.24
BCC892	6	25.13	66.8	6.216	52.76	118.2	22.51
BCC893	6	23.95	71.49	6.337	43.8	134.3	20.67
BCC899	2	26.72	74.32	5.489	49.37	126.3	23.73
BCC900	6	21.37	56.84	4.559	33.77	70.5	16.85
BCC903	2	23.38	66.73	5.984	43.91	123.1	20.5
BCC907	2	28.1	70.78	5.61	46.21	133	24.47
BCC913	2	26.26	56.92	5.97	47.38	141.4	23.57
BCC921	6	26.03	54.34	6.095	55.52	121.2	20.09
BCC927	6	24.31	79.6	5.774	46.45	118	20.47
BCC929	2	22.47	76.44	5.732	54.62	138.8	24.54
BCC942	6	25.36	70.09	5.61	44.9	133.6	24.16
HOR11370	2	25.51	73.61	5.61	43.81	123.3	22.52
HOR11371	2	26.71	71.74	5.281	47.64	111.3	22.26
HOR11372	2	24.76	72.2	6.216	43.84	143.8	22.67
HOR11373	2	25.23	64.42	6.216	43.93	119.3	18.88
HOR11374	2	26.76	65.84	5.557	46.99	102.4	18.91
HOR11403	6	23.87	52.8	5.419	34.47	87.9	16.88
HOR12830	6	26.86	62.3	5.489	49.05	120.4	22.51
HOR1391	2	26.58	62.77	6.337	46.1	129.6	20.02
HOR1804	6	25.87	68.44	5.974	48.89	124.9	20.82
HOR1842	6	26.19	71.49	6.216	51.49	119.5	18.94
HOR1962	6	24.17	59.47	5.853	38.6	103.6	17.71
HOR2800	6	31.15	69.37	5.489	45.05	140.6	26.26
HOR2828	2	26.25	65.13	6.216	50.24	139	23.21
HOR2829	2	28.53	71.49	6.822	59.75	159.7	22.3
HOR2835	6	27.75	77.14	6.108	56.95	132.5	21.18
HOR4727	6	29.82	84.64	5.732	53.12	127.3	22.33
HOR7985	2	29.91	62.3	6.458	55.03	148.3	21.89
HOR8006	2	30.44	57.35	6.337	53.62	152.5	23.55
HOR8050	2	27.35	71.49	5.732	41.86	124.1	21.8
HOR8113	2	27.57	56.65	5.61	44.87	123.8	22.48
HOR8160	2	28.49	64.42	6.458	55.36	170.5	25.24

Table S2. Comparison of root phenotypes for 2-rowed and 6-rowed barley subpopulations. RSD, Root system depth; RSA, Root spreading angle; SRN, Seminal root number; TSRL, Total seminal root length; ASRL, Average seminal root length; SDW, Shoot dry weight.

Traits	Subset	Genotype No.	Min	Max	Mean	SD	CV(%)	*P*<0.05
RSD	2-rowed	125	20.76	31.58	26.14	3.31	12.65	b
	6-rowed	96	8.15	34.03	24.86	5.62	22.61	a
RSA	2-rowed	125	46.67	85.83	66.25	14.15	21.35	a
	6-rowed	96	46.67	131.67	76.02	21.14	27.81	b
SRN	2-rowed	125	4.50	8.00	6.07	0.87	14.40	b
	6-rowed	96	4.22	6.83	5.70	0.87	15.31	a
TSRL	2-rowed	123	102.33	170.43	131.19	24.50	18.68	b
	6-rowed	96	64.56	148.63	116.10	26.28	22.64	a
ASRL	2-rowed	123	17.80	29.95	21.76	3.38	15.53	b
	6-rowed	96	14.59	28.63	20.55	4.18	20.31	a
SDW	2-rowed	125	34.75	70.13	48.83	10.92	22.36	b
	6-rowed	96	18.05	61.28	43.37	11.77	27.14	a

Table S3. Correlations among barley root and shoot traits and TKW. Spearman rank correlation coefficient *r* is presented if *p*<0.05; ns, non-significant. RSD, Root system depth; RSA, Root spreading angle; SRN, Seminal root number; TSRL, Total seminal root length; ASRL, Average seminal root length; SDW, Shoot dry weight; TKW, thousand kernel weight.

Traits	RSD	RSA	SRN	TSRL	ASRL	SDW	TKW
RSD	1	ns	ns	0.40	0.50	0.22	0.24
RSA		1	-0.15	ns	ns	-0.13	-0.24
SRN			1	0.52	-0.24	0.30	0.19
TSRL				1	0.59	0.49	0.42
ASRL					1	0.31	0.31
SDW						1	0.37
TKW							1

Table S4, List of QTLs identified for root traits in the spring barley collection with marker information and trait effects of particular alleles. RSD, Root system depth; RSA, Root spreading angle; SRN, Seminal root number; TSRL, Total seminal root length; ASRL, Average seminal root length; SDW, Shoot dry weight.

QTL	Chr	Position (cM) [a]	$-\log_{10}P$	*q*-value	Associated marker	R^2(%)	Allele [b]	Allele effect [c]	Major allele (count)	TKW [d]	DFL [e]	PHT [f]	CPC [g]	SC [h]	Reference QTL (cM) [i]	Associated trait [j]	Reference
*q*RSD1	1H	17.78	3.12	0.24	SCRI_RS_3336	0.59	C/A	-1.8	A (153)	-1.4	1.4	3.3	-0.2	0.5	15.1	Leaf area	Alqudah et al. 2018
*q*RSD2	2H	18.91	3.11	0.24	SCRI_RS_233272	0.88	T/G	-1.2	G (151)	-0.3	3.4	3.0	-1.1	2.1	18.9-19.9<br>19.9	Phenology<br>Tiller number	Alqudah et al. 2014<br>Alqudah et al. 2016
*q*RSD3	2H	56.52	5.39	0.02	SCRI_RS_220718	12.34	A/G	4.2	G (203)	6.2	5.0	3.2	-2.1	2.8	63.53 (58.8)<br>63.5-66.8 (57-60.7)<br>59.2-63.5 (56.4-57)<br>63.5-64.2 (58.8-59.1)<br>63.53 (57-58)<br>58<br>50-58<br>50.9-56.4	Thousand kernel weight<br>Heading date<br>Plant height<br>Starch content<br>Crude protein content<br>Phenology<br>Tiller number<br>Leaf area	Pasam et al. 2012<br>Pasam et al. 2012<br>Pasam et al. 2012<br>Pasam et al. 2012<br>Pasam et al. 2012<br>Alqudah et al. 2014<br>Alqudah et al. 2016<br>Alqudah et al. 2018
*q*RSD4	2H	126.77	3.47	0.17	SCRI_RS_155734	3.97	T/C	1.4	C (175)	0.8	3.1	6.1	-1.3	3.3	131.77 (124.9)	Plant height	Pasam et al. 2012
*q*RSD5	3H	49.71	3.67	0.11	BOPA2_12_30680	2.74	A/G	2.6	G (184)	6.0	2.6	-2.3	-1.1	2.0	47.09 (46)<br>36.4-46.3 (44.4-45.8)<br>47.09-55.57 (46)<br>46.2	Thousand kernel weight<br>Plant height<br>Crude protein content<br>Tiller number	Pasam et al. 2012<br>Pasam et al. 2012<br>Pasam et al. 2012<br>Alqudah et al. 2016
*q*RSD6	3H	62.25	4.11	0.06	SCRI_RS_174419<br>BOPA2_12_30754	4.84	A/G	2.1	G (144)	4.6	2.7	-3.5	-0.8	1.4	72.26 (62.4)<br>64.3<br>61.8	Crude protein content<br>Phenology<br>Tiller number	Pasam et al. 2012<br>Alqudah et al. 2014<br>Alqudah et al. 2016
*q*RSD7	4H	26.77	3.67	0.11	BOPA1_4616-503	0.17	A/G	2.2	G (161)	6.6	2.9	-2.8	-1.0	2.0	26.66 (26.8)<br>26.3	Thousand kernel weight<br>Phenology	Pasam et al. 2012<br>Alqudah et al. 2014
*q*RSD8	4H	43.63	4.27	0.06	BOPA2_12_10347	0.22	A/G	3.1	G (199)	6.5	4.6	4.0	-2.2	2.7	40.36 (43.8)<br>43.63<br>35.9-45.7	Thousand kernel weight<br>Shoot fresh biomass<br>Tiller number	Pasam et al. 2012<br>Neumann et al. 2017<br>Alqudah et al. 2016
*q*RSD9	4H	51.73	3.96	0.08	SCRI_RS_225722	3.67	A/G	3.6	G (192)	6.7	1.4	-8.7	-0.2	1.5	48.65-53.47<br>51.1-51.6<br>43.3-54.6	Shoot dry weight<br>Phenology<br>Tiller number	Reinert et al. 2016<br>Alqudah et al. 2014<br>Alqudah et al. 2016
*q*RSD10	5H	122.57	4.48	0.06	SCRI_RS_130992	5.78	A/G	2.6	G (160)	1.6	0.7	-6.4	0.3	0.1	132.63 (122.4)<br>114-.7-125.8<br>122.4<br>118.6-118.8	Plant height<br>Phenology<br>Tiller number<br>Leaf area	Pasam et al. 2012<br>Alqudah et al. 2014<br>Alqudah et al. 2016<br>Alqudah et al. 2018

*q*RSD11	5H	135.83	3.71	0.11	SCRI_RS_105705	0.09	A/G	2.6	G (182)	2.3	0.5	1.6	-0.7	0.8	137.2<br>136.8<br>129.4-135.3	Phenology<br>Tiller number<br>Leaf area	Alqudah et al. 2014<br>Alqudah et al. 2016<br>Alqudah et al. 2018
*q*RSD12	5H	146.11	4.32	0.06	BOPA1_5126-1311	2.97	A/G	2.6	G (206)	-1.8	-1.8	2.3	0.7	-1.1	149.5<br>143.7-146.1	Phenology<br>Tiller number	Alqudah et al. 2014<br>Alqudah et al. 2016
*q*RSD13	6H	55.38	3.54	0.13	SCRI_RS_171997	4.54	A/G	1.9	G (126)	1.1	-1.3	-5.0	0.7	-1.0	55.94 (55.67)<br>55-59<br>48.9-55.8<br>59.4-71	Thousand kernel weight<br>Phenology<br>Tiller number<br>Leaf area	Pasam et al. 2012<br>Alqudah et al. 2014<br>Alqudah et al. 2016<br>Alqudah et al. 2018
*q*RSD14	7H	17.53	3.28	0.21	BOPA1_6093-572	0.93	G/A	2.2	A (188)	1.4	1.9	2.8	-0.7	1.6	13.95<br>13.9-20.8<br>17.6<br>11.8-18	Shoot fresh biomass<br>Phenology<br>Tiller number<br>Leaf area	Neumann et al. 2017<br>Alqudah et al. 2014<br>Alqudah et al. 2016<br>Alqudah et al. 2018
*q*RSD15	7H	65.44	4.20	0.06	BOPA1_3186-1560	2.77	A/G	-0.9	G (141)	3.2	3.1	0.9	-0.9	2.4	73-75 (67.8)<br>64-70.5<br>68.4 (62.4)	Plant height<br>Tiller number<br>Heading date	Pasam et al. 2012<br>Alqudah et al. 2016<br>Pasam et al. 2012
*q*RSD16	7H	134.20	3.25	0.21	SCRI_RS_120015	0.63	C/T	-2.1	T (190)	1.9	2.2	7.9	-1.3	2.9	143.68 (131)<br>133.9<br>133.9-140	Thousand kernel weight<br>Biomass yield<br>Leaf area	Pasam et al. 2012<br>Wehner et al.. 2015<br>Alqudah et al. 2018
*q*RSA1	1H	12.46	3.03	0.16	BOPA1_5768-469	0.86	G/A	-11.0	A (158)	3.6	3.2	0.6	-1.4	3.5	15.1	Leaf area	Alqudah et al. 2018
*q*RSA2	1H	55.95	3.24	0.22	BOPA2_12_30950	0.05	G/A	-15.1	A (207)	4.4	2.3	4.4	-1.6	2.9	60.19-69.53 (57-66.3)<br>43.1-55.7<br>59.1-66.3	Starch content<br>Tiller number<br>Leaf area	Pasam et al. 2012<br>Alqudah et al. 2016<br>Alqudah et al. 2018
*q*RSA3	2H	50.04	4.19	0.03	BOPA2_12_10219	1.23	A/G	-17.1	G (202)	3.3	3.4	7.8	-2.3	2.7	40.8-52.8<br>50.0-52.9	Tiller number<br>Leaf area	Alqudah et al. 2016<br>Alqudah et al. 2018
*q*RSA4	2H	58.92	4.59	0.03	BOPA1_3576-2715<br>SCRI_RS_134925<br>SCRI_RS_141789<br>SCRI_RS_153880<br>BOPA2_12_30108	1.68	A/C	-13.6	A (206)	5.9	2.9	2.9	-1.7	3.3	63.53 (58.8)<br>63.5-66.8 (57-60.7)<br>59.2-63.5 (56.4-57)<br>63.5-64.2 (58.8-59.1)<br>63.53 (57-58)<br>58<br>50-58	Thousand kernel weight<br>Heading date<br>Plant height<br>Starch content<br>Crude protein content<br>Phenology<br>Tiller number	Pasam et al. 2012<br>Pasam et al. 2012<br>Pasam et al. 2012<br>Pasam et al. 2012<br>Pasam et al. 2012<br>Alqudah et al. 2014<br>Alqudah et al. 2016
*q*RSA5	3H	45.40	4.98	0.02	SCRI_RS_151254<br>SCRI_RS_227793<br>BOPA2_12_30064	6.38	A/C	-13.2	C (170)	4.9	4.1	7.2	-2.1	4.0	47.09 (46)<br>36.4-46.3 (44.4-45.8)<br>47.09-55.57 (46)<br>45.8<br>46.2<br>49.6-51.6	Thousand kernel weight<br>Plant height<br>Crude protein content<br>Phenology<br>Tiller number<br>Leaf area	Pasam et al. 2012<br>Pasam et al. 2012<br>Pasam et al. 2012<br>Alqudah et al. 2014<br>Alqudah et al. 2016<br>Alqudah et al. 2018

*q*RSA6	3H	67.92	6.50	0.00	BOPA2_12_20849	18.35	A/G	-22.5	G (205)	5.7	4.6	6.3	-2.3	4.0	61.9-68.2	Leaf area	Alqudah et al. 2018
*q*RSA7	3H	86.33	4.40	0.03	BOPA2_12_30090	0.46	A/G	-18.9	G (203)	5.6	3.6	2.0	-2.0	3.4	98.49 (86.3)	Thousand kernel weight	Pasam et al. 2012
*q*RSA8	3H	120.67	3.89	0.05	BOPA2_12_10122	0.18	A/G	-21.8	G (209)	4.2	4.2	4.9	-2.5	3.3	120.68-124.54	Root dry weight	Reinert et al. 2016
*q*RSA9	3H	128.61	3.53	0.09	SCRI_RS_201224	1.00	A/G	-10.4	G (140)	4.1	2.2	0.5	-1.3	2.7	128-137.7	Tiller number	Alqudah et al. 2016
*q*RSA10	4H	21.18	3.77	0.06	BOPA2_12_30394	1.17	A/G	-13.1	G (206)	3.0	3.2	9.6	-2.3	3.6	20.9	Phenology	Alqudah et al. 2014
*q*RSA11	4H	78.40	3.71	0.07	BOPA1_4051-1101	1.94	G/C	-12.6	C (179)	5.0	1.7	-2.5	-0.9	2.5	79.6<br>82.4 (78.8)<br>80.79 (78.4)<br>81.2-91.3	Shoot and root dry weight<br>Heading date<br>Plant height<br>Tiller number	Long et al.. 2013<br>Pasam et al. 2012<br>Pasam et al. 2012<br>Alqudah et al. 2016
*q*RSA12	5H	23.61	3.17	0.18	BOPA2_12_30531	5.98	A/G	-18.8	G (208)	7.4	3.2	2.9	-2.4	3.5	21.3-23.6	Plant height	Alqudah et al. 2016
*q*RSA13	5H	43.76	3.68	0.07	BOPA2_12_10899	1.51	G/A	-12.8	A (183)	0.6	2.8	7.9	-1.3	3.3	51.3 (44.5)<br>44-48<br>46.3-47.5<br>41.3-50	Plant height<br>Phenology<br>Tiller number<br>Leaf area	Pasam et al. 2012<br>Alqudah et al. 2014<br>Alqudah et al. 2014<br>Alqudah et al. 2018
*q*RSA14	5H	60.49	3.07	0.21	BOPA2_12_21157	0.14	G/A	-11.9	A (203)	1.8	2.8	5.3	1.3	1.8	59.7<br>62.6	Biomass yield<br>Leaf area	Wehner et al.. 2015<br>Alqudah et al. 2018
*q*RSA15	5H	125.76	4.02	0.05	BOPA2_12_30867	0.34	A/C	-1.0	C (207)	5.6	4.6	5.5	-1.8	1.9	142.2 (129.4)<br>132.63 (122.4)<br>114-.7-125.8<br>122.4<br>129.4-135.3	Thousand kernel weight<br>Plant height<br>Phenology<br>Tiller number<br>Leaf area	Pasam et al. 2012<br>Pasam et al. 2012<br>Alqudah et al. 2014<br>Alqudah et al. 2016<br>Alqudah et al. 2016<br>Alqudah et al. 2018
*q*RSA16	7H	69.56	3.56	0.09	BOPA2_12_31203	0.07	A/G	-0.5	G (196)	3.8	3.8	5.1	-2.1	4.0	70.2<br>73-75 (67.8)<br>78.22 (71.2)<br>80.94 (71.2)<br>70.5-76.4<br>70.6<br>67.7-71	Biomass yield<br>Plant height<br>Starch content<br>Crude protein content<br>Phenology<br>Tiller number<br>Leaf area	Wehner et al.. 2015<br>Pasam et al. 2012<br>Pasam et al. 2012<br>Pasam et al. 2012<br>Alqudah et al. 2014<br>Alqudah et al. 2016<br>Alqudah et al. 2018
*q*SRN1	2H	148.06	3.04	0.61	SCRI_RS_206020	9	T/C	-0.3	C (158)	1.4	0.5	-2.2	-0.6	0	141.5-147.5<br>146.4-147.5	Phenology<br>Plant height	Alqudah et al. 2014<br>Alqudah et al. 2016
*q*SRN2	3H	135.62	4.33	0.25	SCRI_RS_205957	3	T/G	0.1	G (154)	-0.9	-1.4	3.0	0	-0.5	133<br>137.7	Phenology<br>Tiller number	Alqudah et al. 2014<br>Alqudah et al. 2016
*q*TSRL1	1H	90.44	3.46	0.37	SCRI_RS_213675	0.42	T/C	-9.7	C (128)	-4.2	-3.0	-1.0	0,9	-2.6	92.2<br>92.3<br>90.3-100.7	Biomass yield<br>Phenology<br>Leaf area	Wehner et al.. 2015<br>Alqudah et al. 2014<br>Alqudah et al. 2018

*q*TSRL2	1H	118.56	3.06	0.54	BOPA1_4978-1030	3.23	A/G	-1.1	G (198)	-2.0	1.4	-4.0	-0.1	-1.0	122.09-122.17<br>126.01 (119.1)<br>116.8-119.1	Root dry weight<br>Starch content<br>Phenology	Reinert et al. 2016<br>Pasam et al. 2012<br>Alqudah et al. 2014
*q*TSRL3	2H	76.20	6.50	0.00	SCRI_RS_4930	22.16	T/C	14.3	C (125)	5.9	4.2	0.3	-1.3	3.1	74.4<br>73.7-83.8	Phenology<br>Tiller number	Alqudah et al. 2014<br>Alqudah et al. 2016
*q*TSRL4	2H	136.05	3.12	0.54	SCRI_RS_175216	4.92	T/C	-12.6	C (162)	-3.5	-0.6	2.2	0.3	-0.7	135.6	Leaf area	Alqudah et al. 2018
*q*TSRL5	3H	75.21	4.42	0.06	SCRI_RS_155763	1.78	G/A	14.2	A (127)	3.9	3.2	2.0	-1.2	2.7	76.2<br>75.2	Biomass yield<br>Phenology	Wehner et al.. 2015<br>Alqudah et al. 2014
*q*TSRL6	4H	25.71	3.71	0.26	BOPA1_3687-271	0.42	G/C	13.7	C (117)	5.6	3.9	-0.4	-1.4	3.6	26.66 (26.8)<br>26.3	Thousand kernel weight<br>Phenology	Pasam et al. 2012<br>Alqudah et al. 2014
*q*ASRL1	1H	90.44	3.00	0.45	BOPA1_2935-1634	0.16	C/G	1.0	G (118)	-4.5	-4.2	0.7	1.3	-3.3	92.3<br>81-87	Phenology<br>Leaf area	Alqudah et al. 2014<br>Alqudah et al. 2018
*q*ASRL2	2H	74.08	3.97	0.23	BOPA2_12_31293<br>SCRI_RS_4930<br>SCRI_RS_235860	10.09	A/T	0.1	T (138)	5.6	4.0	0.3	-1.3	3.1	74.4<br>73.7-80.9	Phenology<br>Tiller number	Alqudah et al. 2014<br>Alqudah et al. 2016
*q*ASRL3	2H	136.83	3.17	0.45	SCRI_RS_161636	2.81	A/G	-1.4	G (158)	-3.6	-1.4	0.8	0.5	-1.1	147.94 (137.7)<br>135.6	Crude protein content<br>Leaf area	Pasam et al. 2012<br>Alqudah et al. 2018
*q*ASRL4	4H	25.71	3.67	0.28	BOPA1_3687-271	1.34	G/C	0.08	C (118)	5.6	3.9	-0.4	-1.4	3.6	26.66 (26.8)<br>26.3	Thousand kernel weight<br>Phenology	Pasam et al. 2012<br>Alqudah et al. 2014
*q*ASRL5	4H	48.65	3.17	0.45	SCRI_RS_144322	0.58	A/G	-0.1	G (126)	-4.6	-2.6	1.2	1.0	-2.2	35.9-45.7<br>51.1	Tiller number<br>Tiller number	Alqudah et al. 2016<br>Alqudah et al. 2016
*q*ASRL6	5H	46.32	3.00	0.45	BOPA2_12_10264	1.53	A/G	-0.7	G (204)	-2.5	-0.2	10.1	-0.5	1.1	46.7<br>51.3 (44.5)<br>58.7-65.49 (47.2-51.5)<br>44-48<br>46.3-47.5<br>41.3-50	Biomass yield<br>Plant height<br>Starch content<br>Phenology<br>Tiller number<br>Leaf area	Wehner et al.. 2015<br>Pasam et al. 2012<br>Pasam et al. 2012<br>Alqudah et al. 2014<br>Alqudah et al. 2016<br>Alqudah et al. 2018
*q*ASRL7	6H	95.04	3.14	0.45	BOPA2_12_31042	4.57	C/G	-1.4	G (187)	-0.9	2.5	3.3	-0.9	1.6	88.6-95<br>95.0-95.6	Tiller number<br>Leaf area	Alqudah et al. 2016<br>Alqudah et al. 2018
*q*ASRL8	7H	1.63	4.10	0.23	BOPA2_12_20016	6.45	A/G	-1.8	G (190)	-0.4	-0.1	-0.9	0.2	0.3	3.82<br>0.2-2.1	Root length<br>Leaf area	Reinert et al. 2016<br>Alqudah et al. 2018
*q*SDW1	1H	50.85	4.23	0.31	BOPA2_12_10198	12.70	A/G	5.6	G (139)	5.3	3.6	1.5	-1.4	2.8	51.23-55.49 (48.9)<br>55.49 (48.9)<br>50.4-55.7	Starch content<br>Crude protein content<br>Leaf area	Pasam et al. 2012<br>Pasam et al. 2012<br>Alqudah et al. 2018
*q*SDW2	1H	90.44	3.34	0.66	SCRI_RS_197263	4.93	G/A	5.8	A (121)	4.8	1.8	2.4	-1.0	2.6	92.3	Phenology	Alqudah et al. 2014

*q*SDW3	2H	120.75	3.06	0.66	BOPA1_6652-209	0.05	G/C	5.3	C (162)	6.8	1.9	-2.6	-0.7	2.0	131.77 (124.9)<br>119.8<br>118-120	Plant height<br>Phenology<br>Leaf area	Pasam et al. 2012<br>Alqudah et al. 2014<br>Alqudah et al. 2018
*q*SDW4	3H	154.15	3.10	0.66	BOPA2_12_10981	4.97	C/A	-8.2	A (209)	-3.4	2.3	3.2	-0.8	1.7			
*q*SDW5	4H	51.40	3.00	0.66	BOPA1_ABC14026-1-2-168	1.70	G/A	-4.4	A (194)	-2.4	-0.1	2.1	-0.5	1.2	48.65-53.47<br>51.1-51.6	Shoot dry weight<br>phenology	Reinert et al. 2016<br>Alqudah et al. 2014
*q*SDW6	5H	44.17	3.00	0.66	SCRI_RS_156086	2.80	G/A	3.1	A (154)	-0.9	1.5	4.2	-0.2	0.2	51.3 (44.5)<br>48.2<br>46.7<br>46.3-47.5<br>41.3-50	Biomass yield<br>Plant height<br>Phenology<br>Tiller number<br>Leaf area	Wehner et al.. 2015<br>Pasam et al. 2012<br>Alqudah et al. 2014<br>Alqudah et al. 2016<br>Alqudah et al. 2018
*q*SDW7	7H	23.02	3.13	0.66	SCRI_RS_47197	1.98	G/A	-4.0	A (115)	-1.7	-0.7	0.6	-0.4	-0.8	20.8-24.2<br>23<br>21.3-23.7	Phenology<br>Tiller number<br>Leaf area	Alqudah et al. 2014<br>Alqudah et al. 2016<br>Alqudah et al. 2018

[a] Position of peak marker for each QTL reported.

[b] Marker given as minor allele/major allele.

[c] The allele effect was computed as difference between the means of groups harboring the major allele and the minor allele.

[d-h] Difference in thousand kernel weight (TKW, g), heading date (DFL, days), plant height (PHT, cm), crude protein content (CPC, %) and starch content (SC, %) between groups harboring major and minor alleles. Phenotypic data were obtained from Pasam et al. (2012)

[i] Reference QTLs are reported either as position of the SNP marker or as chromosomal interval; numbers in parenthesis denote the location projected on the POPSEQ map.

[j] Associated traits reported in previous work.

Table S5. Summarized QTL regions associated with multiple traits in the spring barley collection. RSD, Root system depth; RSA, Root spreading angle; SRN, Seminal root number; TSRL, Total seminal root length; ASRL, Average seminal root length; SDW, Shoot dry weight.

QTL cluster	QTL	Chr	Position (cM)	$-\log_{10} P$	*q*-value	Associated markers
Region 1	qSDW2	1H	90.44	3.34	0.66	SCRI_RS_197263
	qTSRL1	1H	90.44	3.46	0.37	SCRI_RS_213675
	qASRL1	1H	90.44	3.00	0.45	BOPA1_2935-1634
Region 2	qRSD3	2H	56.52	5.39	0.02	SCRI_RS_220718
	qRSA4	2H	58.92	4.59	0.03	BOPA1_3576-2715.SCRI_RS_134925.SCRI_RS_141789. SCRI_RS_153880.BOPA2_12_30108
Region 3	qASRL2	2H	74.08	3.97	0.23	BOPA2_12_31293.SCRI_RS_4930.SCRI_RS_235860
	qTSRL3	2H	76.20	6.50	0.00	SCRI_RS_4930
Region 4	qTSRL4	2H	136.05	3.12	0.54	SCRI_RS_175216
	qASRL3	2H	136.83	3.17	0.45	SCRI_RS_161636
Region 5	qRSA5	3H	45.40	4.98	0.02	SCRI_RS_151254.SCRI_RS_227793.BOPA2_12_30064
	qRSD5	3H	49.71	3.67	0.11	BOPA2_12_30680
Region 6	qTSRL6	4H	25.71	3.71	0.26	BOPA1_3687-271
	qASRL4	4H	25.71	3.67	0.28	BOPA1_3687-271
	qRSD7	4H	26.77	3.67	0.11	BOPA1_4616-503
Region 7	qASRL5	4H	48.65	3.17	0.45	SCRI_RS_144322
	qSDW5	4H	51.40	3.00	0.66	BOPA1_ABC14026-1-2-168
	qRSD9	4H	51.73	3.96	0.08	SCRI_RS_225722
Region 8	qRSA13	5H	43.76	3.68	0.07	BOPA2_12_10899
	qSDW6	5H	44.17	3.00	0.66	SCRI_RS_156086
	qASRL6	5H	46.32	3.00	0.45	BOPA2_12_10264
Region 9	qRSD10	5H	122.57	4.48	0.06	SCRI_RS_130992
	qRSA15	5H	125.76	4.02	0.05	BOPA2_12_30867
Region 10	qRSD15	7H	65.44	4.20	0.06	BOPA1_3186-1560
	qRSA16	7H	69.56	3.56	0.09	BOPA2_12_31203

Table S6. Candidate genes identified according to quantitative trait loci (QTL) associated with root traits in barley.

Chromosome	Candidate gene	Accession number	Position (cM)	QTL	Contig identifier
1H	Auxin signaling F-box 2	AK355927	48.23	qRSA2/*q*SDW1	morex_contig_1557974
1H	HvHXK1	MLOC_57896.1	48.08		morex_contig_42192
1H	Gibberellin 2-oxidase 5	AK357218	48.16		morex_contig_5934
1H	HvSuT4	AJ534445.1	49.85		morex_contig_38718
1H	HvGID1-like	AK074026	55.52		morex_contig_137029
1H	HvHXK5	HM037131.1	90.43	*q*SDW2/*q*TSRL1/*q*ASRL1	morex_contig_38242
1H	Ppd-H2/HvFT3	HM133570.1	92.35		morex_contig_2551337
1H	HvGA2ox4	AY551432.1	94.75		morex_contig_1566970
1H	HvARF4 (Auxin response factor 4)	AK357487	92.36		morex_contig_2549990
2H	Ppd-H1	AY970701.1	18.91	*q*RSD2	morex_contig_94710
2H	HvFT4	DQ411320.1	50.04	*q*RSA3/*q*RSD3/*q*RSA4	morex_contig_6666
2H	HvCEN	JX844786.1	58		morex_contig_274284
2H	HvSUSIBA2	AK369730	58.05		morex_contig_206649
2H	HvGID2	MLOC_61457.1	58.78		morex_contig_41142
2H	HvSHR1	MLOC_62665.1	58.06		morex_contig_46826
2H	HvARF6 (Auxin response factor 6)	MLOC_64596.1	58.93		morex_contig_49046
2H	ARF15 (Auxin response factor 15)	AK364144	74.08	*q*TSRL3/*q*ASRL2	morex_contig_52667
2H	Gibberellin 3-beta-hydroxylase	MLOC_12855.1	135.77	*q*ASRL3/*q*TSRL4	morex_contig_1564273
3H	HvHXK7	MLOC_12451.3	45.82	*q*RSD5/*q*RSA5	morex_contig_1563318
3H	HvGA3ox2	MLOC_12855.1	46.03		orex_contig_51542
3H	HvD18	MLOC_12855.1	46.03		morex_contig_51542
3H	HvBRI1	MLOC_5176.2	51.34		morex_contig_58772
3H	HvFT2	AK373041	52.03		morex_contig_1558556
3H	IAA18	MLOC_56819.1	45.83		morex_contig_41178
3H	Auxin response factor	MLOC_65945.1	49.72		morex_contig_50920

3H	HvHXK6	MLOC_54094.1	62.53	*q*RSD6/*q*RSA6	morex_contig_38880
3H	HvMAX4/HvCCD8	MLOC_66551.1	62.93		morex_contig_51744
3H	HvGA2ox1	AK364775	64.16		morex_contig_2550522
3H	Auxin efflux carrier family protein-like	AK357058	76.35	*q*TSRL5	morex_contig_1561160
4H	HvINT-C (Teosinte branched 1)	MLOC_70116.1	25.85	*q*RSD7/*q*RSA10/*q*TSRL6/*q*ASRL4	morex_contig_5747
4H	ARF5 (Auxin response factor 5)	MLOC_11014.7	51.41	*q*RSD9/*q*ASRL5/*q*SDW5	morex_contig_1560200
4H	HvSCR1	AK365059	51.41		morex_contig_86833
4H	HvRHT3	MLOC_52864.1	52.3		morex_contig_37868
5H	HvCPD	MLOC_10658.1	44.02	*q*RSA13/*q*ASRL6/*q*SDW6	morex_contig_1559549
5H	HvDRO1	MLOC_3895.5	48.38		morex_contig_134254
5H	VRN-H1	AK360697	125.76	*q*RSA15	morex_contig_116492
5H	Gibberellin receptor GID1. putative	MLOC_74158.1	137.5	*q*RSD11	morex_contig_65261
6H	auxin response factor 4	MLOC_47091.1	50.93	*q*RSD13	morex_contig_300523
6H	Auxin response factor 18	MLOC_66152.2	52.87		morex_contig_51161
6H	Nitrilase	MLOC_12498.3	54.75		morex_contig_1563418
6H	Auxin response factor 8	MLOC_77438.2	54.89		morex_contig_75658
6H	Nitrilase 2	MLOC_63646.1	55.39		morex_contig_47802
6H	HvTPS2	AK366091	95.04	*q*ASRL7	morex_contig_1581754
7H	*HvKAO1*	MLOC_54352.1	1.9	*q*ASRL8	morex_contig_39067
7H	Hexose transporter	AK354614	23.31	*q*SDW7	morex_contig_243735
7H	HvAFB5 (auxin F-box protein 5)	MLOC_66474.2	61.76	*q*RSD15/*q*RSA16	morex_contig_51607
7H	HvPIN1	MLOC_12686.1	65.76		morex_contig_1563877
7H	Gibberellin receptor GID1L2	MLOC_71630.1	61.76		morex_contig_60206

7H	Cytokinin oxidase/dehydrogenase 3	AK355215	132.65	qRSD16	morex_contig_64780

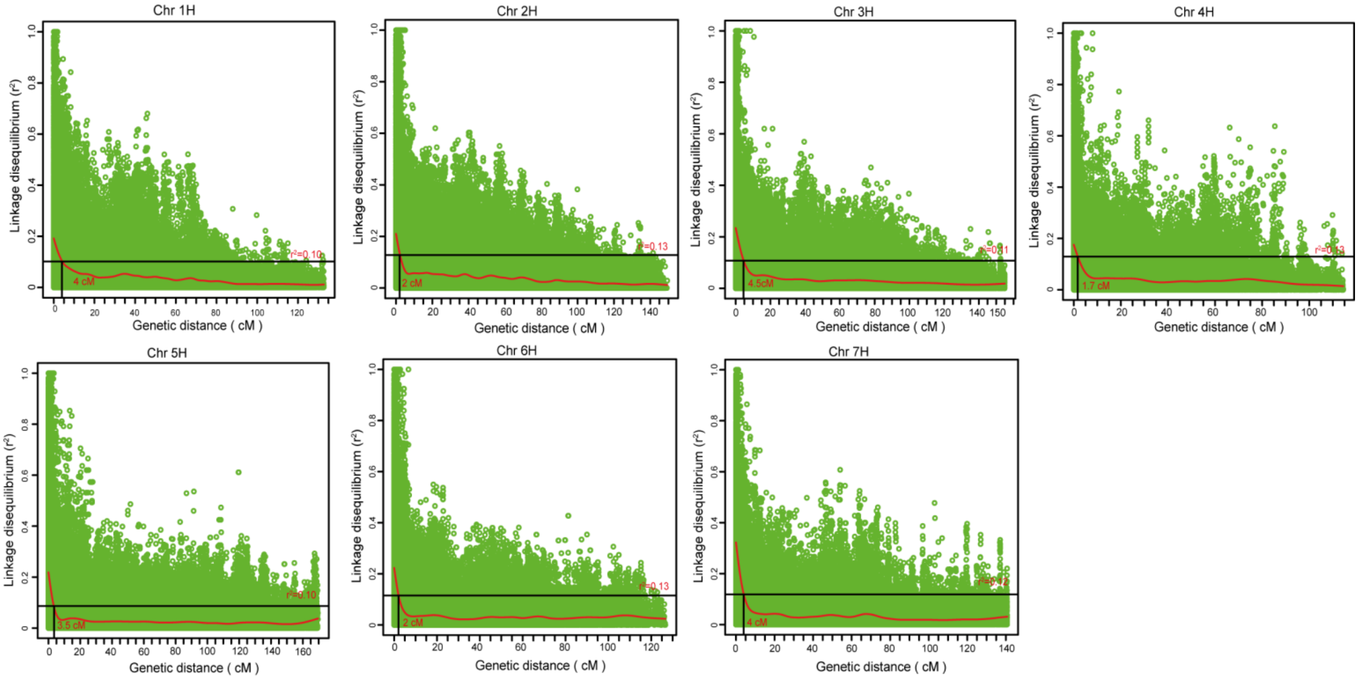

Figure S1. LD decay for each chromosome in the spring barley panel. The rate of LD decay was assessed using the SNP markers genetically mapped to different genomes.The x-axis represents the genetic distance (cM) and the y-axis the association between SNP markers (r^2). The horizontal line indicates the 95th percentile distribution of unlinked r^2. The loess fitting curve (red line) illustrates the LD decay.

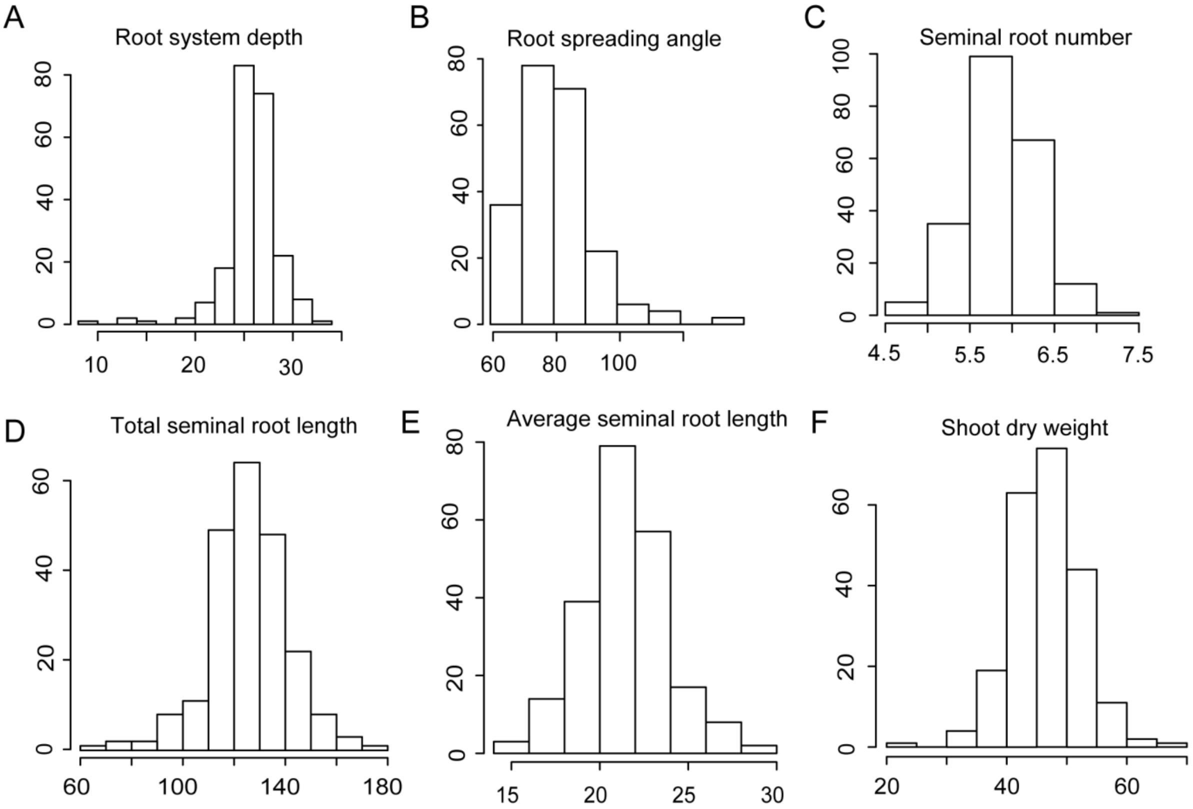

Figure S2. Frequency distribution of root traits measured in the spring barley collection. (A) Root system depth (cm); (B) Root spreading angle (degree); (C) Seminal root number (no,/plant); (D) Total seminal root length (cm); (E) Average seminal root length (cm); (F) Shoot dry weight (g).
